# Vector status of *Aedes* species determines geographical risk of autochthonous Zika virus establishment

**DOI:** 10.1371/journal.pntd.0005487

**Published:** 2017-03-24

**Authors:** Lauren Gardner, Nan Chen, Sahotra Sarkar

**Affiliations:** 1 School of Civil and Environmental Engineering, University of New South Wales (UNSW) Sydney, Sydney, NSW, Australia; 2 Department of Integrative Biology and Department of Philosophy, University of Texas at Austin, Austin, Texas, United States of America; Brandeis University, UNITED STATES

## Abstract

**Background:**

The 2015-16 Zika virus pandemic originating in Latin America led to predictions of a catastrophic global spread of the disease. Since the current outbreak began in Brazil in May 2015 local transmission of Zika has been reported in over 60 countries and territories, with over 750 thousand confirmed and suspected cases. As a result of its range expansion attention has focused on possible modes of transmission, of which the arthropod vector-based disease spread cycle involving *Aedes* species is believed to be the most important. Additional causes of concern are the emerging new links between Zika disease and Guillain-Barre Syndrome (GBS), and a once rare congenital disease, microcephaly.

**Methodology/principal findings:**

Like dengue and chikungunya, the geographic establishment of Zika is thought to be limited by the occurrence of its principal vector mosquito species, *Ae. aegypti* and, possibly, *Ae. albopictus*. While *Ae. albopictus* populations are more widely established than those of *Ae. aegypti*, the relative competence of these species as a Zika vector is unknown. The analysis reported here presents a global risk model that considers the role of each vector species independently, and quantifies the potential spreading risk of Zika into new regions. Six scenarios are evaluated which vary in the weight assigned to *Ae. albopictus* as a possible spreading vector. The scenarios are bounded by the extreme assumptions that spread is driven by air travel and *Ae. aegypti* presence alone and spread driven equally by both species. For each scenario destination cities at highest risk of Zika outbreaks are prioritized, as are source cities in affected regions. Finally, intercontinental air travel routes that pose the highest risk for Zika spread are also ranked. The results are compared between scenarios.

**Conclusions/significance:**

Results from the analysis reveal that if *Ae. aegypti* is the only competent Zika vector, then risk is geographically limited; in North America mainly to Florida and Texas. However, if *Ae. albopictus* proves to be a competent vector of Zika, which does not yet appear to be the case, then there is risk of local establishment in all American regions including Canada and Chile, much of Western Europe, Australia, New Zealand, as well as South and East Asia, with a substantial increase in risk to Asia due to the more recent local establishment of Zika in Singapore.

## Author summary

Between 1952, when the Zika virus was first found in humans, and 2007 Zika disease outbreaks were limited to small isolated epidemics in equatorial Africa and tropical Asia. However, the recent outbreak, which began in Brazil in May 2015, resulted over 750 thousand estimated cases and confirmed local transmission in more than 60 countries by October, 2016. Like dengue and chikungunya, Zika is spread by *Aedes aegypti* mosquitoes and possibly, other species including *Aedes albopictus*. Geographic spread of the virus occurs when infected travelers travel from affected regions to ones without an established local Zika disease cycle, but in which the known and potential vector species have established populations. We estimate the risk of Zika importation and establishment into new regions using air travel data and ecological vector habitat suitability models for *Ae. aegypti* and *Ae. albopictus*. Given the uncertainties surrounding the vectorial competence of *Aedes* mosquitoes, we compare the geographic risk profiles when spread is driven by air travel and *Ae. aegypti* presence alone, with spread driven by air travel and both species. We conclude that there is a much higher global risk of Zika spread under the latter scenario, although it is the least likely.

## Introduction

In May 2015, a Zika disease outbreak originated in Brazil, and by October 5, 2016, local transmission of the Zika virus had been reported in over 60 countries and territories, with over 750 thousand estimated cases [1]. The World Health Organization (WHO) previously predicted that the virus would establish itself in all countries in the Americas except Canada and Chile [2], and with few exceptions this scenario has proved true. Travel-imported cases have also been increasingly reported throughout the United States, as well as in Australia, New Zealand, Canada, Western Europe, and China [3], representing the first time Zika has been reported in many of these western countries.

The Zika virus was first isolated in 1947 in a sentinel monkey in the Zika forest of Uganda [4] which gave the virus its name. It was first found in humans in 1952 [5]. However, only 14 human cases were documented prior to 2007 [6], and these were limited to small isolated epidemics in equatorial Africa and tropical Asia [6]. Since the 1950s the virus has spread eastwards from Africa through Asia and the Pacific, culminating with the 2015-16 outbreak in Latin America [7, 8].

The first documented recent outbreak of Zika disease occurred on Yap Island in the Federated States of Micronesia in the North Pacific in 2007 with less than 200 acknowledged cases [6]. In 2013 another outbreak occurred in French Polynesia, with around 28,000 suspected cases, at which point Zika began to be generally recognized as a re-emerging infectious disease [9]. The outbreak subsequently spread from French Polynesia to other Pacific Islands including New Caledonia, Cook Island, and Easter Island, where autochthonous transmission cycles were established [10]. Travel–imported cases were also documented in Japan [11], Germany [12] and Norway [10], among other regions.

The recent outbreak in Latin America began in Brazil, with the first documented Zika case reported in May, 2015 [13], although phylogenetic analyses of virus RNA sequences suggest that the virus was introduced into the Americas between May and December 2013 [14]. The virus quickly spread from Brazil throughout Latin America; by February 2016, an estimated 31,555 cases were identified in Colombia alone [2].

Historically, Zika infection has been associated with mild symptoms typically resembling and milder than those of related arboviruses such as dengue and chikungunya; many cases of infection show no symptoms at all. However, the recent outbreaks in French Polynesia and Latin America have been associated with much more serious clinical manifestations of the virus. In Brazil and French Polynesia, a link between Zika and a rare congenital disease, microcephaly, has been identified [15–22]. Additionally, Guillain-Barre Syndrome (GBS) has been reported in patients infected with Zika, firstly in the 2013 French Polynesia outbreak [23], and since in greater numbers in Brazil, El Salvador, Venezuela, Colombia, and Suriname [22]. The unprecedented size of the outbreak, rate of spread, and potential links with microcephaly and GBS prompted the WHO to declare the current Zika virus outbreak a public health emergency of international concern [24].

Zika now joins a list of arboviral diseases such as dengue and chikungunya that are being increasingly reported in new parts of the world, all likely introduced through global transport systems such as passenger air travel and maritime freight [14, 25]. Geographic spread of the virus occurs when infected travelers travel from affected regions to ones without local establishment of the disease, but in which the known and suspected vector species have established populations. Like dengue and chikungunya, Zika is known to be spread by *Aedes aegypti*; it is also strongly suspected to be spread by *Aedes albopictus*. While vectorial competence of *Ae. aegypti* is well established [26–29], and it is now confirmed to be the primary vector in spreading Zika [30–32], the capacity of *Ae. albopictus* as a secondary vector for spreading Zika is still unclear. There is evidence of the potential role of *Ae. albopictus* [33, 34], however, there is limited and conflicting quantitative estimate of its efficiency [35, 36]. Jupille *et. al.* [35] found that both *Ae. aegypti* from Madeira Island and *Ae. albopictus* from France were able to transmit the Zika virus, however *Ae. albopictus* from France was found to be less suitable to sustain local transmission. Chouin-Carneiro *et. al.* [36] observed high infection but low transmission rates for both *Ae. aegypti* and *Ae. albopictus*, while WHO [31] notes the vector competence for both species is similar, but *Ae. albopictus* is considered to have lower vector capacity than *Ae. aegypti*. The outcomes from these studies suggests both species are capable of Zika transmission, while also highlighting the uncertainty in the role that *Ae. albopictus* may play in transmitting, spreading, and helping to maintain the virus in many areas of the world. Further, potential virus adaptation to new vectors, as demonstrated in the case of Chikungunya in La Reunion [37, 38], introduces additional uncertainties.

The uncertainty surrounding the vectorial competence of different *Aedes* species in spreading Zika serves as the main motivation for the present analysis. This study explicitly addresses the differences in the potential geographical risk of Zika spread and local disease cycle establishment if *Ae. aegypti* is the sole competent vector versus if both *Ae. aegypti* and *Ae. albopictus* are competent for this purpose. Scenarios which further vary in the relative capacity of *Ae. albopictus* as a secondary vector are also considered.

As noted earlier, available evidence indicates that the two species differ in their vectorial competence. Moreover, the two species also vary widely in their present geographic distribution: *Ae. aegypti* is mainly present in wet tropical regions, while *Ae. albopictus*, a much better disperser, has a wider global presence in temperate regions, including the northern United States and parts of Canada, southern regions of the Americas including Chile, parts of Western Australia and East Asia. The analysis presented here is global and carried out at the finest resolution (1 arc-minute) that was permitted by the available data. Some preliminary findings were reported earlier [39] but the methodology was not described; all the analyses have been expanded and updated here and expectations from those preliminary findings were used to validate conclusions from the analysis using “back-testing”.

Several recent studies have mapped the potential spread of Zika into new regions [40–43]. These studies differ from the present one in either the assumptions made about the competence of potential vector species, in the spatial resolution or geographical extent of the study areas used, or in the methodological tools that were used. Monaghan *et. al.* [42] simulated *Ae. aegypti* and *Ae. albopictus* mosquito abundance based on meteorological models, and overlaid the results with travel and socioeconomic factors to estimate the cities in the United States with the highest expected cases of travel-imported Zika. Nah *et. al.* [43] presented a global risk model for Zika importation which used survival analysis and publicly available epidemiological and air travel data to predict the risk of importation and local transmission of Zika at the country level. In one study, Bogoch *et. al.* [40] presented an air travel-based risk map of Zika spread from Brazil into the rest of the Americas, and in another study modeled risk posed to Africa and the Asia Pacific region [41]. Both works [40, 41] implicitly assumed Zika to be equally efficiently spread by both *Ae. aegypti* and *Ae. albopictus*, and all studies only considered airline travelers departing the Americas. However, on August 28, 2016 local Zika spread was confirmed in Singapore, and autochthonous Zika transmission has since been reported across multiple community clusters [44]. With Singapore serving as a new potential source of Zika infected travelers, a substantially higher risk is posed to South and South-east Asia, where *Aedes* mosquito populations are well established, and Zika and dengue are endemic.

The present analysis extends previous work by presenting a global risk analysis based on a new mathematical framework to estimate Zika importation and establishment risk at a city level based on the most recent state of the outbreak, and accounting for uncertainties regarding the vectorial competence of *Ae. albopictus*. The risk analysis reported in this paper considers six scenarios, A, B, C, D, E and F, respectively, which vary in their assumed relative capacity of *Ae. albopictus* compared to *Ae. aegypti*, as a secondary spreading vector of Zika. The scenarios are bounded by two extreme assumptions; in Scenario A spread is assumed to be driven by *Ae. aegypti* presence alone, while in Scenario F spread is driven by *Ae. aegypti* and *Ae. albopictus* presence equally. In Scenarios B through E spread is driven predominately by *Ae. aegypti* presence with *Ae. albopictus* presence playing a lesser role. These scenarios are further described in the Materials and Methods section.

Besides air travel data, this work utilizes ecological vector habitat suitability models for *Ae. aegyti* and *Ae. albopictus* previously developed to analyze the role of air travel in the risk of geographical spread of dengue [45–47]. Those models are relevant to the risk of Zika spread because the same two vector species are implicated with one difference: while *Ae. aegypti* is known to be an efficient vector for both diseases, in the case of dengue *Ae. albopictus* is also known to be a competent but less efficient vector, whereas in the case of Zika it is a likely vector but its relative competence is unknown. Thus, the focus of this analysis will be on four questions:

1. For each scenario, what is the *expected relative risk posed to unaffected regions* by Zika-infected travelers arriving (by air) from known affected regions?
2. For each scenario, what is the *expected relative risk posed by airports in affected regions* by Zika-infected travelers departing (by air) to unaffected regions?
3. What are the intercontinental air travel routes that pose the highest risk of spreading Zika into new regions?
4. To what extent did *Ae. albopictus* play a secondary role in the 2015-2016 Zika pandemic in Latin America?

The analysis reported here only considers potential vectorial transmission of Zika. It ignores other modes of transmission that have been reported including sexual transmission [22] and congenital transmission [22].

## Materials and methods

Throughout this analysis, the expected relative risk was computed as a function of three main components, the volume of travelers moving between Zika-affected regions and unaffected regions, the probabilistic expectation of established vector populations at the origin, and the probabilistic expectation of established vector populations at the destination. Because of the role of air travel, airports are central to this analysis as both origins and destinations for the spread of Zika. The vector suitability models and travel statistics used to compute passenger travel volumes are explained in further detail in the Data Section.

### Risk model

The following protocol was used for a scenario specific risk analysis. Six scenarios are considered, A–F, which vary in the assumed relative capacity of *Ae. albopictus* as a spreading vector of Zika. The six scenarios are bounded by Scenario A, where spread is assumed driven by air travel and *Ae. aegypti* presence alone, and Scenario F, where spread is assumed to be driven by air travel and both species equally. Scenario B, C, D and E represent cases where *Ae. albopictus* plays a secondary role to *Ae. aegypti*. Specifically, the relative capacity of *Ae. albopictus* compared with *Ae. aegypti* ranges from 10% to 75% across these scenarios. Assigned weights are used in the six scenarios to represent the relative capacity, and are *w* = 0, 0.10, 0.25, 0.5, 0.75 and 1 for Scenarios A–F, respectively. In Scenario A the assigned weight is 0, representing the case where *Ae. albopictus* has no capacity to spread Zika, while in Scenario F the assigned weight is equal to 1.0, representing equal capacity for the two species. The range of weights is selected to demonstrate the variability in the risk posed to or from a particular location as a function of the relative capacity of *Ae. albopictus* to transmit Zika. Because *Ae. aegypti* has been confirmed as the primary spreading vector of Zika, the sensitivity analysis is more focused on the lower relative capacities of *Ae. albopictus*, which is suspected to play a much lesser role. Given these six scenarios, the protocol consists of seven stages:

1. Identify the complete set of source airports, *S*, as those in countries in which autochthonous Zika transmission has been reported as of October 5, 2016 according to the Centers for Disease Control and Prevention (CDC) [3].
2. Define the set of potentially at-risk destination airports *D*, as those airports located in cities in unaffected regions as of October 5, 2016 according to the CDC [3].
3. Define $\sigma_{i}^{k}$, the relative habitat suitability for each airport *i* for each scenario *k* as a function of *Ae. aegypti* and *Ae. albopictus* established vector presence in each location, and the scenario specific weight, *w*. For Scenario *k*, the relative habitat suitability in the city served by airport *i*,is defined as:
  $$\sigma_{i}^{k}=1-\left[\left(1-\sigma_{i}^{ae}\right)\left(1-w*\sigma_{i}^{alb}\right)\right]$$ (1)
  where $\sigma_{i}^{ae}$ is the relative habitat suitability of *Ae. aegypti* in the city served by airport *i*, $\sigma_{i}^{alb}$ is the relative habitat suitability of *Ae. albopictus* in the city served by airport *i*, and *w* is the weight assigned in scenario *k*, ranging between 0 and 1. When *w* = 0, $\sigma_{i}^{k}=\sigma_{i}^{ae}$, or simply the expected presence of *Ae. aegypti* in the city served by airport *i*. When *w* = 1, $\sigma_{i}^{k}$ is equivalent to the expected presence of either *Ae. aegypti* or *Ae. albopictus* in the city served by airport *i*. The habitat suitabilities for each vector are normalized to a 0-1 scale.
4. For each scenario *k*, compute the travel route risk for each origin-destination pair, *ij*, connecting a source airport, *i*, and an at-risk destination airport, *j*. Define the route risk as
  $$r_{ij}^{k}=V_{ij}\sigma_{i}^{k}\sigma_{j}^{k}$$ (2)
  where *V*_*ij*_ is the volume of travel between origin airport *i* ∈ *S* and destination airport *j* ∈ *D*, and $\sigma_{i}^{k}$ is the relative vector habitat suitability for scenario *k* in the city served by airport *i*.
5. For each scenario *k*, compute the destination risk posed to each at-risk airport *j*, $d_{j}^{k}$, by aggregating the incoming route risks from all source airports in set *S*,
  $$d_{j}^{k}=\sum_{i\in S}r_{ij}^{k}\;\forall j\in D$$ (3)
6. For each scenario *k*, compute the origin risk posed by each source airport *i*, $o_{i}^{k}$, by aggregating the outgoing route risks across all connected at-risk destination airports in set *D*,
  $$o_{i}^{k}=\sum_{j\in D}r_{ij}^{k}\;\forall i\in S$$ (4)
7. For steps 5 and 6, the computed risks for each origin and destination for each scenario were normalized to the highest risk value in Scenario D as follows: ${\tilde{d}}_{j}^{k}=\frac{d_{j}^{k}}{max_{j}d_{j}^{F}}\;\forall j\in D$ and ${\tilde{o}}_{i}^{k}=\frac{o_{i}^{k}}{max_{i}o_{i}^{F}}\;\forall i\in S$, respectively, to reflect the expected relative risk (a value ranging between 0 and 1) posed in each scenario.

### Back-testing

A separate analysis was conducted to evaluate the performance of the risk model. The protocol described above was re-implemented, wherein the set of source airports, *S*, was defined as those in areas with autochthonous Zika transmission as of February 15, 2016 rather than October 5, 2016 [39]. Between February 15 and October 5, 2016, 29 new countries and territories were added to the CDC list of affected regions. The ranking and relative risk quantified by the proposed model for each scenario for these 29 countries is presented and discussed. This analysis also serves to identify the sceanrio most consistent with the observed outbreak, and thus the role played by the secondary spreading vector, *Ae. albopictus*.

### Data

The proposed risk model is based on data from the global air traffic network and species distribution models for the principle spreading vectors species.

#### Travel data

The transportation data was obtained from the International Air Transport Association (IATA), and included origin, destination and stopover airports for all routes, as well as the calibrated passenger travel volumes for each route. The route-specific passenger travel volumes supplied by IATA were calibrated based on data from 240 airlines comprising 84% of global air traffic, and includes over 3400 airports. The passenger volumes were available at a monthly temporal resolution, which thus determined the temporal resolution of the model. The transportation data used in this paper were limited to passenger travel volumes and did not include cargo flights on which vectors could potentially be transported because the latter mode of Zika spread was excluded from this model. The analysis was done using flight paths and travel volumes for all routes in October 2015. These are the most recent data available for this annual period.

#### Species distribution models

The risk for the establishment of Zika and potential cases of disease in an originally non-endemic area depends fundamentally on the ability of a vector to establish itself in that area, that is, on the ecological conditions for the vector there. When these ecological conditions are suitable, the disease may become endemic if the vector species successfully disperses to that area. A quantitative relative measure of the suitability of one area compared to another defines the relative ecological risk of that area [48–50]. If the ecological risk is low, such an establishment is highly unlikely. If that risk is high, then other factors, such as the (temporally) immediate ambient environmental conditions and the size of the founder population or the availability of hosts, become critical for establishment.

This analysis was based on habitat suitability for the two principal Zika vector species, *Ae. aegypti* and *Ae. albopictus*. The relative ecological risk for the establishment for each species was estimated using a global species distribution model at a 1 arc-minute resolution [51, 52] based on a maximum entropy algorithm incorporated in the Maxent software package [53, 54]. Details of these models have been published earlier in the context of risk of dengue spread through air travel [45–47]. The same algorithm has since been used by others to model the distribution of these species at a coarser spatial resolution [55]. A different algorithm has been used by another group, also at a coarser resolution, to map the global distribution of the two vectors [56], and the environmental suitability for Zika [57]. While it is impossible to compare these results formally because of the differences in spatial resolution, the predicted distributions all appear to share the same geographical extent suggesting strongly that these models are good indicators of where these *Aedes* species are likely to be present.

In this risk analysis, it was assumed that these two species do not interact, that is, the probability of the presence of each is independent of that of the presence of the other. Thus, the probabilistic expectation of at least one of the vector species being present in a cell was calculated as the complement of the probability that neither is present, assuming statistical independence, as defined in eq (1). The expectations are aggregated to the city level by averaging them over all the cells in the relevant geographical units. These expectations define the relative ecological risk for Zika in each cell. Habitat suitability for each city in this analysis was computed by aggregating across all cells in a circle with a radius of 50 km. This study does not explicitly account for population size or the human-to-vector ratio in a region, although the arrival travel volumes, which are included, are highly correlated to regional population levels.

## Results

**Fig 1:**
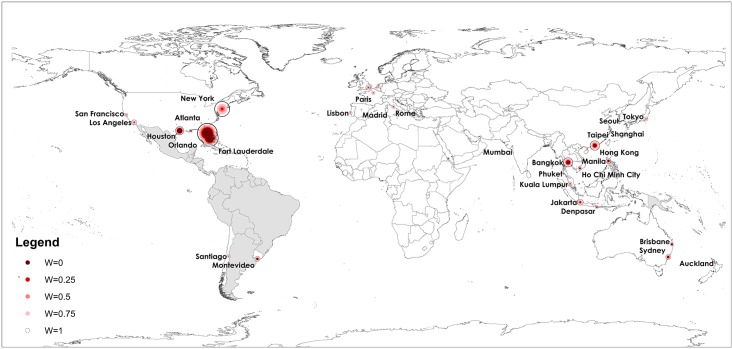
Destination Risk Map for Spread of Zika. The circles depict the top 100 cities to which Zika may be imported from source regions, resulting in local outbreaks. The countries shaded in gray are those with reported autochthonous Zika transmission as of October 5, 2016. The size of the circle is the estimated relative risk, with the color indicative of the scenario. The darkest shade indicates the risk from *Ae. aegypti* alone. Lighter color circles indicate total risk from both species, weighted according to scenarios C, D, E and F.

The destination risk model results are illustrated in Fig 1. The top 100 cities to which Zika may be imported from affected regions for scenarios A, C, D, E and F are shown. The results for Scenario B are too hard to distinguish from A and C in the figure, so it is left out. The size of the circle represents the estimated expected relative risk posed to each city, with the color indicating the scenario. For those cities which are served by more than one international airport, the relative risk for all airports which serve the given city are aggregated. Solid dark red indicates the risk from *Ae. aegypti* alone, *i.e.*, Scenario A, and the color of the circles lightens progressively from Scenario A to Scenario F. All risk values are computed using eq (3), for their respective scenarios.

S1 Table contains the list of the top 100 at risk destination cities included in the map for all six scenarios, including their corresponding rank, relative risk, and designated country.

To gain a better understanding of the risk posed by outgoing travelers, the risk posed by each city in a known affected region for exporting infected travelers is also assessed. The top 100 origin cities in the affected regions likely to export Zika to new regions are listed in S2 Table, including their corresponding rank, relative risk, and designated country. Similarly to the destination risk, the relative risk at the city level is aggregated over all airports which serve a given city.

S3 and S4 Tables further breaks down the previous results to identify those routes which carry the most risk into and out of cities, and include the top 100 highest risk origin-destination city travel pairs for Scenario A and F, respectively.

Finally, in regard to validation, the model was run for each scenario using the set of sources as of February 15, 2016 along with travel data for February 2015. The destination risk results were aggregated to the country level, ranked and compared with the actual set of 29 counties/territories that were added to the CDC list of confirmed affected regions between February 15th, 2016 and October 5, 2016. Results from the back-testing analysis for each scenario are presented in S5 and S6 Tables. S5 Table lists the set of 29 Countries with local Zika transmission confirmed between February 15 and October 5 and the relative ranking for each of those countries computed for each of the six scenarios. S6 Table lists the top 29 countries at risk for the six scenarios. The results reveal Scenario A identified more of the 29 countries in it’s top 29 ranking than the other three scenarios, however all scenarios identified at least 15 of the 29, in their top 29. A more detailed discussion of these results will be presented as part of the Discussion below.

## Discussion

By October 5, 2016 Zika had already become established throughout most of the Americas, which was also predicted by our earlier analysis [39]. Based on the status of the outbreak in early October 2015, the results reveal the United States and South and Southeast Asia to be at greatest risk of Zika becoming locally established. This is true for all scenarios considered, although the top 100 set of cities and corresponding rank does vary among the six scenarios. The main differences being if *Ae. aegypti* is the only competent Zika vector, then risk of local establishment in north America is concentrated in Texas and Florida, with South and East Asia and Australia following closely. However, if *Ae. albopictus* is a competent vector of Zika, then there is increased risk of local establishment in much of Western Europe and the U.S., Canada, Chile, Australia, New Zealand, as well as South and East Asia. In total, the risk posed to each country is increased by at least a factor of three, when both species of mosquitoes are considered.

The destination cities at highest risk are similar across all scenarios, with Orlando, Fort Lauderdale, Houston, Bangkok and Hong Kong topping all lists. As the assumed role of *Ae. albopictus* increases across the scenarios, the risk posed to major cities in the U.S. such as New York City and Los Angeles increases, as does the risk to major cities in Western Europe, specifically Rome, London, Paris and Amsterdam. When *Ae. albopictus* is considered an equal spreading vector, New York City becomes one of the top five at risk destinations, outranking Houston. The high risk posed to cities in Florida, Houston and New York is predominately due to the recent establishment of Zika in Miami. In fact, the Miami-to-Houston travel route is ranked in the top ten of all travel routes for Scenario A; however, it is replaced by the Miami-to-New York travel route for Scenario F. Within the U.S. our results are also consistent with those from [42]; both studies conclude Texas and Florida are at highest risk, specifically Miami, Orlando and Houston. Increasingly, travel cases are being reported in almost all U.S. States, and other countries including Argentina, Chile, France, Italy, New Zealand [22], which are all identified to be at high risk by our model.

The more recent increased risk posed to cities in Asia is predominately due to the local establishment of Zika in Singapore. Singapore is a global travel hub, with especially high connectivity to major cities throughout the Asia Pacific region, which also have established populations of *Aedes* mosquitoes, and are prone to outbreaks of dengue and even some small outbreaks of Zika. For this reason, cities in Thailand, Hong Kong, Indonesia, the Philippines, China, India, Taiwan, Vietnam and Malaysia are at high risk of importation resulting in local outbreaks. In fact, in September 2016 the CDC issued a Zika travel notice to 11 Zika endemic countries in Southeast Asia after confirmed local outbreaks in the region, however, the actual risk posed to locals and travelers remains unclear. Cities in Australia and New Zealand are similarly at increased risk due to the high travel connectivity with the Asia-Pacific region. For the scenarios which consider additional risk posed by *Ae. albopictus*, Tokyo, Japan as well as multiple European cities including Rome, London, Paris, Amsterdam, and Lisbon, and even San Francisco are identified to be at risk of local establishment. Of the 20 highest ranked cities in Scenario F, four are served by at least two major airports, including Tokyo, London, Paris, and New York, which partially contributes to their increased risk of Zika importation and establishment, as illustrated in Fig 1. The destinations at highest risk of Zika importation should prepare and implement mosquito surveillance and control efforts, in order to reduce the likelihood of local establishment, or, in those Southeast Asian cities where Zika is already endemic, the likelihood of new local outbreaks.

An additional means to control the spread of Zika is to conduct surveillance at the sources of travel to limit the number of outgoing cases. However, it is unrealistic to implement control at every airport in a region affected by Zika. By having a better understanding of the risk posed by individual airports and cities the available resources in a country can be optimally allocated. For all scenarios Singapore is identified as the highest risk origin. This is again due to its highly connected and globally central location. Miami, Florida, San Juan, Puerto Rico, and Cancun, Mexico are ranked second, third, and fourth in all scenarios, with Nadi, Fiji and Nassau, Bahamas ranking 5th and 6th in scenarios A, B and C, and Sao Paulo, Brazil ranking 5th in Scenario D, E and F. This outcome is of particular concern because many of these cities are highly popular tourist destinations for many U.S. and European residents. In order to prevent further global spread and establishment of Zika, it is imperative that these major travel destinations get the local outbreaks under control.

In efforts to better understand the destination and origin rankings discussed previously, the city-level analysis was further disaggregated to explicitly identify the travel routes carrying the highest risk of spreading Zika into new regions. This type of analysis allows for the specific incoming and outgoing flights which pose the highest risk to be identified and targeted for mosquito surveillance and targeted passenger screening. As noted above, these route-level results are included in S3 and S4 Tables, for Scenario A and F only. For these two scenarios, six and eight of the highest risk routes originate in Singapore. When only *Ae. aegypti* is considered, six of the top ten highest risk routes depart Singapore for cities in south and east Asia, with the top one arriving in Bangkok, followed by Hong Kong. Three of the top ten routes arrive in Florida, from either San Juan or Nassau, and the travel route between Miami and Houston is listed 10th. For Scenario F, eight of the top ten highest risk routes depart Singapore to cities in Asia. The only two routes in the top ten list not departing Singapore are from San Juan to Orlando and Miami to New York. These routes align closely with the top origins and destinations discussed previously.

### Model validation

As the Zika outbreak continues to progress, the number of countries with local transmission is increasing, and this was especially the case during the first half of 2016. The results presented thus far serve as projected relative risk estimates for each city, and can be used to identify the locations most likely to see imported cases followed by local outbreaks in the near future. However, in an attempt to evaluate the model’s ability to accurately identify the regions most likely to experience future outbreaks, as well as identify the level of contribution of *Ae. albopictus* in the outbreak, we implemented the model using the state of the outbreak in February 15, 2016 (to define the set of high risk sources), and compared the model outcomes across all scenarios with the actual set of regions later confirmed to be infected. (These earlier results were partially noted in [39]). In fact, all six scenarios ranked Miami, Florida as the top at-risk destination by a significant margin, and in late July, 2016 the first autochthonous Zika cycle in the United States was reported to have been established in the Miami, Florida region.

Between February 15th, 2016 and October 5, 2016 29 new counties or territories were added to the CDC list of confirmed affected regions. These countries are listed in S5 Table. For each of the six scenarios considered, a country level ranking was computed by aggregating the incoming risk across all cities in a given country, and ranking the countries accordingly. These country-level results from the back-testing are presented in S5 with their respective ranking, alongside the list of new countries added to the CDC list during that time. All six scenarios identified at least 15 of the 29 countries in their respective top ranked 29. However, Scenario A outperformed the other five scenarios, with 21 of the top ranked 29 countries accounted for. As the assumed relative capacity of *Ae. albopictus* increased, the number of top ranked countries matching the 29 confirmed affected countries decreased. This result suggest that Scenario A, which only accounts for *Ae. aegypti* presence, is the most accurate model for identifying the regions most likely to experience local establishment in the future.

However, it is important to recognize the discrepancy in the rankings across scenarios highlights an important factor; when comparing the performance of the different scenarios it is important to distinguish between risk of importation and risk of local establishment, the later of which we are comparing the results with. In the five scenarios which account for the additional presence of *Ae. albopictus*, an increasing number of countries identified as high risk (these are listed in S6 Table) are in more developed regions, compared with those countries identified by Scenario A. This discrepancy is because suitable habitats for *Ae. albopictus* expand much further north and south of the equator when compared with *Ae. aegypti*, therefore many cities in Europe, as well as Japan, Australia, New Zealand, and major cities in the northern U.S. are at substantially increased risk of Zika establishment only if *Ae. albopictus* is a capable spreading vector. While these locations, critically, have established vector populations and have experienced a high number of imported cases [3], with the sole exception of Miami, they did not lead to local establishment, likely due to the resources available for local mosquito control and surveillance. Therefore, until the capacity, or lack there of, of *Ae. albopictus* is confirmed, the cities identified at highest risk in all Scenarios should continue to be subject to a high level of surveillance.

Finally, the country level risk predictions in [43] are also consistent with the outcomes of this study. After aggregating the city level relative risks to the country level, the United States and Argentina were identified to be at highest risk in the present study. Nah *et. al.* [43] (who excluded the U.S.) also identified Argentina to be at highest risk of Zika importation, followed by Portugal, Uruguay, Spain, and Peru, which are also among our top ranked countries across the scenarios. While many of the same countries were identified to be at high risk by both models, discrepancies in the rankings exists for various reasons. Firstly, Nah *et. al.* [43] estimated the risks of importation and local transmission separately, while our model combines the two within a single risk modeling framework. Secondly, the present study was conducted at a later state in the outbreak when more countries were confirmed to have local transmission; these countries are listed in [43] as at-risk of importation, while in the present study they are considered to pose additional risk. Additionally, the present study is conducted at the city level instead of the country level, and due to the more spatially disaggregate analysis, the results can not be directly compared. Although the methodologies vary substantially between these studies, the consistency among the model results serves to further validate the present study.

The preliminary findings did not identify the Federated States of Micronesia or the Marshall Islands as high at-risk destinations of any rank, which highlights one of the potential limitations of this analysis that will be explicitly discussed below.

### Limitations

This work takes a major step towards improving our understanding of the spreading risk posed by Zika, however there are six limitations of this analysis, including persistent uncertainties regarding epidemiological parameter estimates, which must be noted here and addressed in future research:

1. The back-testing showed that the preliminary finding based on the methodology used in this analysis failed to identify two apparently high risk destinations: the Federated States of Micronesia and the Marshall Islands. The reason for this is a limitation in the available travel data; airports in these countries had no incoming travel from regions with confirmed local cases. One possible explanation for this is that infected travelers’ itineraries are not fully captured in the data set, *i.e.*, they had separate bookings so records of their complete travel history were absent. Such cases could include a traveler who holidayed in Brazil and became infected with Zika, then traveled to California for a few days on one booking, and then traveled to the Marshall Islands on a separate booking. While this passenger clearly represents a risk to the Marshall Islands, the incomplete travel data limits the ability of the model to identify this traveler as a potential source of infection. Real time mobility data from telecoms which tracks individuals over time is one possible means to address this issue. Another possible explanation for the discrepancy is incorrect epidemiological data, *i.e.*, the travel origin (of the individuals who introduced Zika into New Caledonia or Marshall Islands) was actually an affected region which had not yet been recognized as a source risk.
2. A second recognized limitation of this analysis is due to the lack of even more recent travel data. Over the last year global air travel increased on average at a steady rate, however certain regions of the world have grown faster than others [58]. Specifically, Latin America, Asia-Pacific and Middle East regions have seen a slightly higher than average increases in air travel volumes. Furthermore, the computed risk in the analysis is systematically underestimated because flights originating in other areas historically endemic for Zika that are not currently reporting local transmission are not taken into account.
3. Zika could be efficiently spread by mosquito species other than *Ae. aegypti* and even *Ae. albopictus*. Ayres [59] has recently pointed out that the virus has been collected from at least ten mosquito species from the genus *Aedes*, as well as from species from the genera *Anopheles*, *Culex*, and *Mansonia*, while Aliota *et. al.* [60] confirmed neither *Culex pipiens* or *Ae. triseriatus* mosquitoes are competent Zika virus vectors. However, the presence of the virus does not automatically make the species an efficient vector for the disease. The risk analysis strategy that has been used in this analysis can be extended to take these details into account as shown by the risk maps for malaria in Africa produced by Moffett *et al* [49]. As this analysis underscores for *Ae. albopictus*, relative vector competence has a substantial impact on the geographic risk profile, and further research into the parameters relevant to determining vector competencies is still necessary for Zika transmission.
4. This study accounts for a single mode of inter-regional travel, passenger air trips. Global geographical spread is delimited by travel almost entirely, and air travel captures the vast majority of long distance trips. However, shorter distance trips, especially over land, can be made using alternative travel modes such as car and bus, which are excluded from this study. Therefore, the results from this analysis likely underestimate the risk posed to destinations in close spatial proximity to affected regions. An example of this is Cameron County in Texas, where local cases have been confirmed. This county shares a border with Mexico, and there are high volumes of road travel between the two. To accurately capture multi-modal human mobility patterns either requires data from multiple transport sources, or data which is not linked to any specific transport mode, such as mobile phone data, which as noted previously, offers significant potential in modeling and predicting epidemic spreading risk.
5. In this study outbreak size is not explicitly accounted for at the set of sources, and is instead treated as a binary variable, *i.e.*, local transmission is either present or not. This can potentially lead to biases in the model, especially against countries that have substantially high travel volumes, but smaller outbreaks. In such a case the risk posed would be overestimated. This proposed risk analysis could easily be adapted to incorporate outbreak size in terms of cases, however, accurately estimating the number of cases in a given city at a given time at the global scale is, at this point, an unrealistic option. However, this would be a substantially useful data set were the proper public health authority to provide it, and could increase the accuracy of the model predictions.
6. Finally, this analysis ignores the role of potential spreading mechanisms other than human-to-human transmission mediated through a mosquito vector. In addition to the traditional spreading risk posed by mosquito populations, new potential spreading mechanisms have recently emerged for Zika. There have been several verified and potential documented cases of sexual transmission of Zika [61–63]. The virus has also been detected in the saliva [64] and urine [65] of infected individuals, and vertical cases of transmission have been documented in the Pacific Islands [17]. The Zika virus has also been found in non-human primates, suggesting the possibility of maintenance and spread through alternative animal hosts [4, 33, 66–70]. While local establishment may depend on a variety of factors, vector habitat suitability will play a predominant role even though there may be other modes of transmission. Nonetheless, further research is necessary to understand if and where these potential modes of transmission are a factor in the local spreading risk of Zika. How such factors will necessitate modification of this risk analysis remains to be seen.

### Conclusions

Results from this analysis highlight the substantial geographic and quantitative increase in global risk posed as a function of the relative capacity of *Ae. albopictus* as a secondary spreading vector of Zika, and reveal the set of cities at greatest risk of Zika importation and establishment. The results from the back-testing suggest that the geographic spread of Zika is driven primarily by *Ae. aegypti*, which is consistent with other studies [30–32]. However, the results from the different scenarios also highlight the increased risk posed to new parts of the world, specifically the U.S. and Europe, if *Ae. albopictus* were to become a more capable spreading vector. To control the spread of Zika geographically, local surveillance and control efforts are required in both known affected regions and at-risk regions yet to report cases. This is true for locations with reported travel-imported cases that have yet to see locally established cases.

As the Zika outbreak continues to spread internationally, so does the uncertainty surrounding the local transmission mechanisms and clinical manifestations of the disease. The possibility of direct human-to-human Zika transmission demands further immediate investigation, and the link between Zika and microcephaly and GBS are of vital concern. Lastly, the uncertainty associated with Zika risk is further compounded based on the implications from the analysis presented here which shows that the vector competence of *Ae. albopictus* relative to *Ae. aegypti* demands further investigation. This goal can only be achieved through a combination of field studies to collect a representative variety of strains of these vectors followed by laboratory studies of virus transmission.

## Supporting information

S1 Table Top 100 Destination Cities at Risk under all Scenarios. (PDF)

S2 Table Top 100 Origin Cities Posing Risk under all Scenarios. (PDF)

S3 Table Top 100 High Risk Travel Routes under Scenario A. (PDF)

S4 Table Top 100 High Risk Travel Routes under Scenario D. (PDF)

S5 Table Set of Countries with local Zika transmission confirmed between February 15 and October 5 and relative ranking by Scenario. (PDF)

S6 Table Top 29 Countries identified at greatest risk by each scenario based on outbreak status as of February 15, 2016. (PDF)
