# Supplementary material for: Vector status of Aedes species determines geographical risk of autochthonous Zika virus establishment
Source: PLoS Negl Trop Dis. 2017 Mar 24;11(3):e0005487. doi: 10.1371/journal.pntd.0005487 (PMC5381944; doi:10.1371/journal.pntd.0005487)
Supplement: S1 Table — (PDF) [file pntd.0005487.s001.pdf]

## S1 TABLE: Top 100 Destination Cities at Risk under all Scenarios

| | Scenario A | | | Scenario B | | |
|---|---|---|---|---|---|---|
| Ranking | City | Country | Relative Risk | City | Country | Relative Risk |
| 1 | Orlando | United States | 0.5234 | Orlando | United States | 0.5650 |
| 2 | Fort Lauderdale | United States | 0.4503 | Fort Lauderdale | United States | 0.4843 |
| 3 | Houston | United States | 0.2536 | Houston | United States | 0.2681 |
| 4 | Bangkok | Thailand | 0.1747 | Bangkok | Thailand | 0.1979 |
| 5 | Hong Kong | Hong Kong | 0.1524 | Hong Kong | Hong Kong | 0.1768 |
| 6 | Tampa | United States | 0.1174 | Tampa | United States | 0.1259 |
| 7 | Sydney | Australia | 0.1000 | Sydney | Australia | 0.1121 |
| 8 | Manila | Philippines | 0.0920 | Manila | Philippines | 0.1046 |
| 9 | Montevideo | Uruguay | 0.0905 | Montevideo | Uruguay | 0.1034 |
| 10 | Brisbane | Australia | 0.0876 | Brisbane | Australia | 0.0961 |
| 11 | Ho Chi Minh City | Vietnam | 0.0673 | Ho Chi Minh City | Vietnam | 0.0760 |
| 12 | Jakarta | Indonesia | 0.0553 | Jakarta | Indonesia | 0.0718 |
| 13 | Denpasar | Indonesia | 0.0527 | New York | United States | 0.0676 |
| 14 | New Orleans | United States | 0.0465 | Denpasar | Indonesia | 0.0613 |
| 15 | Jacksonville | United States | 0.0314 | New Orleans | United States | 0.0517 |
| 16 | Phuket | Thailand | 0.0314 | Los Angeles | United States | 0.0370 |
| 17 | Guangzhou | China | 0.0269 | Phuket | Thailand | 0.0362 |
| 18 | Hanoi | Vietnam | 0.0264 | Kuala Lumpur | Malaysia | 0.0352 |
| 19 | Madras | India | 0.0261 | Jacksonville | United States | 0.0339 |
| 20 | Kuala Lumpur | Malaysia | 0.0239 | Guangzhou | China | 0.0320 |
| 21 | Auckland | New Zealand | 0.0230 | Auckland | New Zealand | 0.0307 |
| 22 | Taipei | Taiwan | 0.0226 | Hanoi | Vietnam | 0.0296 |
| 23 | Delhi | India | 0.0215 | Madras | India | 0.0289 |
| 24 | Los Angeles | United States | 0.0197 | Taipei | Taiwan | 0.0279 |
| 25 | Surabaya | Indonesia | 0.0197 | London | United Kingdom | 0.0274 |

| | | | | | | |
|---|---|---|---|---|---|---|
| 26 | Mumbai | India | 0.0190 | Rome | Italy | 0.0269 |
| 27 | Dhaka | Bangladesh | 0.0188 | Delhi | India | 0.0233 |
| 28 | San Antonio | United States | 0.0187 | Surabaya | Indonesia | 0.0229 |
| 29 | Charleston | United States | 0.0181 | Mumbai | India | 0.0225 |
| 30 | Yangon | Burma | 0.0173 | Washington | United States | 0.0217 |
| 31 | Austin | United States | 0.0150 | Dhaka | Bangladesh | 0.0213 |
| 32 | Rome | Italy | 0.0147 | San Antonio | United States | 0.0202 |
| 33 | Tucson | United States | 0.0147 | Yangon | Burma | 0.0201 |
| 34 | Honolulu | United States | 0.0145 | Lisbon | Portugal | 0.0200 |
| 35 | Colombo | Sri Lanka | 0.0142 | San Francisco | United States | 0.0199 |
| 36 | Dallas-Fort Worth | United States | 0.0135 | Charleston | United States | 0.0196 |
| 37 | New York | United States | 0.0134 | Santiago | Chile | 0.0183 |
| 38 | Washington | United States | 0.0129 | Tokyo | Japan | 0.0178 |
| 39 | Fort Myers | United States | 0.0129 | Atlanta | United States | 0.0166 |
| 40 | Tallahassee | United States | 0.0126 | Colombo | Sri Lanka | 0.0163 |
| 41 | Cairns | Australia | 0.0116 | Amsterdam | Netherlands | 0.0162 |
| 42 | Sanford | United States | 0.0113 | Austin | United States | 0.0162 |
| 43 | Gainesville | United States | 0.0113 | Paris | France | 0.0161 |
| 44 | Phnom-penh | Cambodia | 0.0113 | Dallas-Fort Worth | United States | 0.0160 |
| 45 | Bandar Seri Begawan | Brunei | 0.0112 | Honolulu | United States | 0.0159 |
| 46 | Tiruchirappalli | India | 0.0110 | Barcelona | Spain | 0.0154 |
| 47 | Pensacola | United States | 0.0106 | Tucson | United States | 0.0153 |
| 48 | Key West | United States | 0.0106 | Newark | United States | 0.0143 |
| 49 | Coolangatta | Australia | 0.0106 | Shanghai | China | 0.0140 |
| 50 | Atlanta | United States | 0.0105 | Fort Myers | United States | 0.0137 |
| 51 | Savannah | United States | 0.0104 | Tallahassee | United States | 0.0137 |
| 52 | Shenzhen | China | 0.0104 | Penang | Malaysia | 0.0132 |
| 53 | Bermuda | Bermuda | 0.0102 | Cairns | Australia | 0.0129 |
| 54 | Lisbon | Portugal | 0.0102 | Phnom-penh | Cambodia | 0.0128 |

| | | | | | | |
|---|---|---|---|---|---|---|
| 55 | Penang | Malaysia | 0.0095 | Bandar Seri Begawan | Brunei | 0.0124 |
| 56 | Port-vila | Vanuatu | 0.0086 | Tiruchirappalli | India | 0.0124 |
| 57 | Barcelona | Spain | 0.0085 | Melbourne | Australia | 0.0121 |
| 58 | Mcallen | United States | 0.0085 | Shenzhen | China | 0.0120 |
| 59 | London | United Kingdom | 0.0084 | Coolangatta | Australia | 0.0120 |
| 60 | Melbourne | Australia | 0.0084 | Gainesville | United States | 0.0119 |
| 61 | Pulau | Malaysia | 0.0079 | Philadelphia | United States | 0.0119 |
| 62 | Krabi | Thailand | 0.0079 | Sanford | United States | 0.0118 |
| 63 | West Palm Beach | United States | 0.0079 | Pensacola | United States | 0.0116 |
| 64 | Phoenix | United States | 0.0079 | Madrid | Spain | 0.0114 |
| 65 | Macau | Macau | 0.0078 | Bermuda | Bermuda | 0.0111 |
| 66 | Kuching | Malaysia | 0.0077 | Savannah | United States | 0.0111 |
| 67 | Santiago | Chile | 0.0076 | Key West | United States | 0.0111 |
| 68 | San Francisco | United States | 0.0076 | Seoul | South Korea | 0.0098 |
| 69 | Cebu | Philippines | 0.0072 | Pulau | Malaysia | 0.0094 |
| 70 | Bangalore | India | 0.0071 | Boston | United States | 0.0093 |
| 71 | Kolkata | India | 0.0069 | Port-vila | Vanuatu | 0.0093 |
| 72 | Kaohsiung | Taiwan | 0.0066 | Krabi | Thailand | 0.0092 |
| 73 | Xiamen | China | 0.0066 | Macau | Macau | 0.0091 |
| 74 | Tarawa | Kiribati | 0.0063 | Mcallen | United States | 0.0089 |
| 75 | El Paso | United States | 0.0063 | Kuching | Malaysia | 0.0086 |
| 76 | Angeles City | Philippines | 0.0062 | West Palm Beach | United States | 0.0083 |
| 77 | Shanghai | China | 0.0061 | Cebu | Philippines | 0.0082 |
| 78 | Madrid | Spain | 0.0058 | Phoenix | United States | 0.0082 |
| 79 | Hat Yai | Thailand | 0.0057 | Bangalore | India | 0.0080 |
| 80 | Philadelphia | United States | 0.0057 | Xiamen | China | 0.0079 |
| 81 | Haikou | China | 0.0057 | Perth | Australia | 0.0077 |
| 82 | Kochi | India | 0.0057 | Baltimore | United States | 0.0077 |
| 83 | Siem-reap | Cambodia | 0.0057 | Kolkata | India | 0.0077 |

| | | | | | | |
|---|---|---|---|---|---|---|
| 84 | Wallis | Wallis and Futuna | 0.0056 | Chicago | United States | 0.0077 |
| 85 | Agana | Guam | 0.0056 | Kaohsiung | Taiwan | 0.0074 |
| 86 | Myrtle Beach | United States | 0.0055 | Seattle | United States | 0.0071 |
| 87 | Corpus Christi | United States | 0.0052 | Angeles City | Philippines | 0.0070 |
| 88 | Tokyo | Japan | 0.0051 | Tarawa | Kiribati | 0.0068 |
| 89 | Lafayette | United States | 0.0044 | El Paso | United States | 0.0066 |
| 90 | Chiang Mai | Thailand | 0.0041 | Hat Yai | Thailand | 0.0065 |
| 91 | Newark | United States | 0.0041 | Haikou | China | 0.0065 |
| 92 | Baltimore | United States | 0.0041 | Kochi | India | 0.0064 |
| 93 | Boston | United States | 0.0041 | Siem-reap | Cambodia | 0.0063 |
| 94 | Chicago | United States | 0.0041 | Toronto | Canada | 0.0060 |
| 95 | Perth | Australia | 0.0040 | Myrtle Beach | United States | 0.0060 |
| 96 | Luanda | Angola | 0.0040 | Agana | Guam | 0.0060 |
| 97 | Shreveport | United States | 0.0039 | Wallis | Wallis and Futuna | 0.0059 |
| 98 | Geralds | Montserrat | 0.0039 | Luanda | Angola | 0.0055 |
| 99 | Brownsville | United States | 0.0039 | Corpus Christi | United States | 0.0055 |
| 100 | Yogyakarta | Indonesia | 0.0038 | Vancouver | Canada | 0.0052 |

| | Scenario C | | | Scenario D | | |
|---|---|---|---|---|---|---|
| Ranking | City | Country | Relative Risk | City | Country | Relative Risk |
| 1 | Orlando | United States | 0.6301 | Orlando | United States | 0.7451 |
| 2 | Fort Lauderdale | United States | 0.5376 | Fort Lauderdale | United States | 0.6321 |
| 3 | Houston | United States | 0.2905 | New York | United States | 0.3302 |
| 4 | Bangkok | Thailand | 0.2350 | Houston | United States | 0.3293 |
| 5 | Hong Kong | Hong Kong | 0.2166 | Bangkok | Thailand | 0.3031 |
| 6 | New York | United States | 0.1574 | Hong Kong | Hong Kong | 0.2921 |
| 7 | Tampa | United States | 0.1391 | Sydney | Australia | 0.1674 |
| 8 | Sydney | Australia | 0.1315 | Montevideo | Uruguay | 0.1633 |
| 9 | Manila | Philippines | 0.1250 | Manila | Philippines | 0.1629 |
| 10 | Montevideo | Uruguay | 0.1243 | Tampa | United States | 0.1625 |
| 11 | Brisbane | Australia | 0.1095 | Jakarta | Indonesia | 0.1566 |
| 12 | Jakarta | Indonesia | 0.1001 | Brisbane | Australia | 0.1338 |
| 13 | Ho Chi Minh City | Vietnam | 0.0898 | Los Angeles | United States | 0.1204 |
| 14 | Denpasar | Indonesia | 0.0752 | London | United Kingdom | 0.1180 |
| 15 | Los Angeles | United States | 0.0656 | Ho Chi Minh City | Vietnam | 0.1151 |
| 16 | New Orleans | United States | 0.0599 | Denpasar | Indonesia | 0.1017 |
| 17 | London | United Kingdom | 0.0586 | Kuala Lumpur | Malaysia | 0.0952 |
| 18 | Kuala Lumpur | Malaysia | 0.0549 | Rome | Italy | 0.0866 |
| 19 | Rome | Italy | 0.0472 | Tokyo | Japan | 0.0855 |
| 20 | Phuket | Thailand | 0.0439 | San Francisco | United States | 0.0791 |
| 21 | Auckland | New Zealand | 0.0431 | Amsterdam | Netherlands | 0.0778 |
| 22 | Guangzhou | China | 0.0406 | Paris | France | 0.0777 |
| 23 | San Francisco | United States | 0.0402 | New Orleans | United States | 0.0749 |
| 24 | Tokyo | Japan | 0.0401 | Santiago | Chile | 0.0721 |
| 25 | Jacksonville | United States | 0.0377 | Lisbon | Portugal | 0.0719 |
| 26 | Amsterdam | Netherlands | 0.0375 | Auckland | New Zealand | 0.0665 |

| | | | | | | |
|---|---|---|---|---|---|---|
| 27 | Paris | France | 0.0373 | Washington | United States | 0.0630 |
| 28 | Lisbon | Portugal | 0.0372 | Newark | United States | 0.0629 |
| 29 | Taipei | Taiwan | 0.0368 | Phuket | Thailand | 0.0584 |
| 30 | Santiago | Chile | 0.0365 | Guangzhou | China | 0.0571 |
| 31 | Washington | United States | 0.0360 | Shanghai | China | 0.0570 |
| 32 | Hanoi | Vietnam | 0.0346 | Taipei | Taiwan | 0.0543 |
| 33 | Madras | India | 0.0334 | Barcelona | Spain | 0.0508 |
| 34 | Newark | United States | 0.0310 | Atlanta | United States | 0.0450 |
| 35 | Mumbai | India | 0.0284 | Jacksonville | United States | 0.0445 |
| 36 | Surabaya | Indonesia | 0.0281 | Hanoi | Vietnam | 0.0438 |
| 37 | Shanghai | China | 0.0280 | Seoul | South Korea | 0.0425 |
| 38 | Barcelona | Spain | 0.0273 | Madras | India | 0.0413 |
| 39 | Atlanta | United States | 0.0265 | Philadelphia | United States | 0.0404 |
| 40 | Delhi | India | 0.0259 | Mumbai | India | 0.0396 |
| 41 | Dhaka | Bangladesh | 0.0253 | Madrid | Spain | 0.0395 |
| 42 | Yangon | Burma | 0.0247 | Surabaya | Indonesia | 0.0381 |
| 43 | San Antonio | United States | 0.0225 | Boston | United States | 0.0341 |
| 44 | Philadelphia | United States | 0.0218 | Yangon | Burma | 0.0334 |
| 45 | Charleston | United States | 0.0218 | Dhaka | Bangladesh | 0.0328 |
| 46 | Madrid | Spain | 0.0209 | Penang | Malaysia | 0.0327 |
| 47 | Seoul | South Korea | 0.0205 | Melbourne | Australia | 0.0305 |
| 48 | Dallas-Fort Worth | United States | 0.0199 | Delhi | India | 0.0304 |
| 49 | Colombo | Sri Lanka | 0.0197 | Seattle | United States | 0.0282 |
| 50 | Penang | Malaysia | 0.0196 | Perth | Australia | 0.0276 |
| 51 | Melbourne | Australia | 0.0184 | Dallas-Fort Worth | United States | 0.0271 |
| 52 | Honolulu | United States | 0.0181 | San Antonio | United States | 0.0267 |
| 53 | Austin | United States | 0.0180 | Colombo | Sri Lanka | 0.0261 |
| 54 | Boston | United States | 0.0179 | Charleston | United States | 0.0258 |
| 55 | Tucson | United States | 0.0161 | Toronto | Canada | 0.0256 |

| | | | | | | |
|---|---|---|---|---|---|---|
| 56 | Tallahassee | United States | 0.0152 | Chicago | United States | 0.0247 |
| 57 | Phnom-penh | Cambodia | 0.0152 | Baltimore | United States | 0.0244 |
| 58 | Cairns | Australia | 0.0151 | Honolulu | United States | 0.0220 |
| 59 | Fort Myers | United States | 0.0150 | Austin | United States | 0.0213 |
| 60 | Shenzhen | China | 0.0147 | Vancouver | Canada | 0.0205 |
| 61 | Tiruchirappalli | India | 0.0146 | Shenzhen | China | 0.0199 |
| 62 | Seattle | United States | 0.0144 | Istanbul | Turkey | 0.0197 |
| 63 | Coolangatta | Australia | 0.0144 | Phnom-penh | Cambodia | 0.0197 |
| 64 | Bandar Seri Begawan | Brunei | 0.0143 | Frankfurt | Germany | 0.0197 |
| 65 | Perth | Australia | 0.0142 | Portland | United States | 0.0194 |
| 66 | Chicago | United States | 0.0136 | Cairns | Australia | 0.0189 |
| 67 | Baltimore | United States | 0.0135 | Porto | Portugal | 0.0187 |
| 68 | Pensacola | United States | 0.0133 | Coolangatta | Australia | 0.0187 |
| 69 | Toronto | Canada | 0.0129 | Tiruchirappalli | India | 0.0186 |
| 70 | Gainesville | United States | 0.0129 | Tallahassee | United States | 0.0181 |
| 71 | Sanford | United States | 0.0126 | Bandar Seri Begawan | Brunei | 0.0176 |
| 72 | Bermuda | Bermuda | 0.0125 | Tucson | United States | 0.0174 |
| 73 | Savannah | United States | 0.0121 | Fort Myers | United States | 0.0172 |
| 74 | Pulau | Malaysia | 0.0119 | Pulau | Malaysia | 0.0166 |
| 75 | Key West | United States | 0.0119 | Milano | Italy | 0.0164 |
| 76 | Krabi | Thailand | 0.0113 | Pensacola | United States | 0.0162 |
| 77 | Macau | Macau | 0.0113 | San Diego | United States | 0.0156 |
| 78 | Vancouver | Canada | 0.0105 | Macau | Macau | 0.0154 |
| 79 | Port-vila | Vanuatu | 0.0102 | Krabi | Thailand | 0.0154 |
| 80 | Xiamen | China | 0.0101 | Bermuda | Bermuda | 0.0151 |
| 81 | Kuching | Malaysia | 0.0099 | Brussels | Belgium | 0.0148 |
| 82 | Cebu | Philippines | 0.0099 | Norfolk | United States | 0.0148 |
| 83 | Portland | United States | 0.0098 | Tel-aviv | Israel | 0.0147 |
| 84 | Frankfurt | Germany | 0.0097 | Gainesville | United States | 0.0145 |

| | | | | | | |
|---|---|---|---|---|---|---|
| 85 | Porto | Portugal | 0.0096 | Xiamen | China | 0.0144 |
| 86 | Mcallen | United States | 0.0096 | Sanford | United States | 0.0140 |
| 87 | Istanbul | Turkey | 0.0094 | Savannah | United States | 0.0140 |
| 88 | Bangalore | India | 0.0094 | Venice | Italy | 0.0133 |
| 89 | West Palm Beach | United States | 0.0089 | Key West | United States | 0.0133 |
| 90 | Kolkata | India | 0.0089 | Luanda | Angola | 0.0133 |
| 91 | Phoenix | United States | 0.0087 | Cebu | Philippines | 0.0129 |
| 92 | San Diego | United States | 0.0086 | Montreal | Canada | 0.0122 |
| 93 | Kaohsiung | Taiwan | 0.0086 | Kuching | Malaysia | 0.0122 |
| 94 | Norfolk | United States | 0.0085 | Port-vila | Vanuatu | 0.0120 |
| 95 | Angeles City | Philippines | 0.0083 | Bangalore | India | 0.0119 |
| 96 | Luanda | Angola | 0.0081 | Raleigh-durham | United States | 0.0117 |
| 97 | Hat Yai | Thailand | 0.0078 | Osaka | Japan | 0.0117 |
| 98 | Milano | Italy | 0.0077 | Duesseldorf | Germany | 0.0111 |
| 99 | Tel-aviv | Israel | 0.0077 | Kolkata | India | 0.0110 |
| 100 | Haikou | China | 0.0077 | Angeles City | Philippines | 0.0108 |

| | Scenario E | | | Scenario F | | |
|---|---|---|---|---|---|---|
| Ranking | City | Country | Relative Risk | City | Country | Relative Risk |
| 1 | Orlando | United States | 0.8684 | Orlando | United States | 1.0000 |
| 2 | Fort Lauderdale | United States | 0.7339 | Fort Lauderdale | United States | 0.8430 |
| 3 | New York | United States | 0.5317 | New York | United States | 0.7619 |
| 4 | Bangkok | Thailand | 0.3793 | Hong Kong | Hong Kong | 0.4767 |
| 5 | Hong Kong | Hong Kong | 0.3788 | Bangkok | Thailand | 0.4633 |
| 6 | Houston | United States | 0.3703 | Houston | United States | 0.4133 |
| 7 | Jakarta | Indonesia | 0.2249 | Jakarta | Indonesia | 0.3050 |
| 8 | Sydney | Australia | 0.2074 | London | United Kingdom | 0.2647 |
| 9 | Montevideo | Uruguay | 0.2073 | Los Angeles | United States | 0.2569 |
| 10 | Manila | Philippines | 0.2056 | Montevideo | Uruguay | 0.2565 |
| 11 | Tampa | United States | 0.1877 | Manila | Philippines | 0.2531 |
| 12 | London | United Kingdom | 0.1867 | Sydney | Australia | 0.2517 |
| 13 | Los Angeles | United States | 0.1842 | Tampa | United States | 0.2146 |
| 14 | Brisbane | Australia | 0.1606 | Tokyo | Japan | 0.2081 |
| 15 | Kuala Lumpur | Malaysia | 0.1448 | Kuala Lumpur | Malaysia | 0.2036 |
| 16 | Ho Chi Minh City | Vietnam | 0.1433 | Brisbane | Australia | 0.1897 |
| 17 | Tokyo | Japan | 0.1415 | Rome | Italy | 0.1859 |
| 18 | Rome | Italy | 0.1328 | Paris | France | 0.1776 |
| 19 | Denpasar | Indonesia | 0.1321 | Amsterdam | Netherlands | 0.1765 |
| 20 | Paris | France | 0.1245 | San Francisco | United States | 0.1758 |
| 21 | San Francisco | United States | 0.1243 | Ho Chi Minh City | Vietnam | 0.1743 |
| 22 | Amsterdam | Netherlands | 0.1241 | Denpasar | Indonesia | 0.1664 |
| 23 | Santiago | Chile | 0.1145 | Lisbon | Portugal | 0.1644 |
| 24 | Lisbon | Portugal | 0.1143 | Santiago | Chile | 0.1638 |
| 25 | Newark | United States | 0.0999 | Newark | United States | 0.1419 |
| 26 | Washington | United States | 0.0940 | Shanghai | China | 0.1363 |

| | | | | | | |
|---|---|---|---|---|---|---|
| 27 | Shanghai | China | 0.0931 | Washington | United States | 0.1289 |
| 28 | Auckland | New Zealand | 0.0930 | Auckland | New Zealand | 0.1228 |
| 29 | New Orleans | United States | 0.0913 | Barcelona | Spain | 0.1124 |
| 30 | Barcelona | Spain | 0.0792 | New Orleans | United States | 0.1092 |
| 31 | Guangzhou | China | 0.0764 | Seoul | South Korea | 0.1015 |
| 32 | Taipei | Taiwan | 0.0751 | Taipei | Taiwan | 0.0992 |
| 33 | Phuket | Thailand | 0.0749 | Guangzhou | China | 0.0986 |
| 34 | Seoul | South Korea | 0.0695 | Phuket | Thailand | 0.0935 |
| 35 | Atlanta | United States | 0.0660 | Atlanta | United States | 0.0894 |
| 36 | Madrid | Spain | 0.0615 | Madrid | Spain | 0.0869 |
| 37 | Philadelphia | United States | 0.0614 | Philadelphia | United States | 0.0847 |
| 38 | Hanoi | Vietnam | 0.0540 | Boston | United States | 0.0740 |
| 39 | Boston | United States | 0.0528 | Mumbai | India | 0.0676 |
| 40 | Mumbai | India | 0.0526 | Penang | Malaysia | 0.0675 |
| 41 | Jacksonville | United States | 0.0518 | Hanoi | Vietnam | 0.0651 |
| 42 | Madras | India | 0.0498 | Perth | Australia | 0.0640 |
| 43 | Surabaya | Indonesia | 0.0495 | Surabaya | Indonesia | 0.0625 |
| 44 | Penang | Malaysia | 0.0486 | Seattle | United States | 0.0618 |
| 45 | Melbourne | Australia | 0.0447 | Melbourne | Australia | 0.0609 |
| 46 | Perth | Australia | 0.0442 | Jacksonville | United States | 0.0597 |
| 47 | Seattle | United States | 0.0440 | Madras | India | 0.0589 |
| 48 | Yangon | Burma | 0.0434 | Toronto | Canada | 0.0558 |
| 49 | Dhaka | Bangladesh | 0.0410 | Yangon | Burma | 0.0547 |
| 50 | Toronto | Canada | 0.0399 | Chicago | United States | 0.0521 |
| 51 | Chicago | United States | 0.0376 | Baltimore | United States | 0.0506 |
| 52 | Baltimore | United States | 0.0367 | Dhaka | Bangladesh | 0.0502 |
| 53 | Dallas-Fort Worth | United States | 0.0353 | Istanbul | Turkey | 0.0471 |
| 54 | Delhi | India | 0.0349 | Vancouver | Canada | 0.0449 |
| 55 | Colombo | Sri Lanka | 0.0333 | Frankfurt | Germany | 0.0445 |

| | | | | | | |
|---|---|---|---|---|---|---|
| 56 | Istanbul | Turkey | 0.0323 | Dallas-Fort Worth | United States | 0.0443 |
| 57 | Vancouver | Canada | 0.0320 | Portland | United States | 0.0427 |
| 58 | Frankfurt | Germany | 0.0313 | Porto | Portugal | 0.0424 |
| 59 | San Antonio | United States | 0.0312 | Colombo | Sri Lanka | 0.0414 |
| 60 | Portland | United States | 0.0304 | Delhi | India | 0.0395 |
| 61 | Charleston | United States | 0.0301 | Milano | Italy | 0.0383 |
| 62 | Porto | Portugal | 0.0297 | San Antonio | United States | 0.0361 |
| 63 | Milano | Italy | 0.0266 | Charleston | United States | 0.0348 |
| 64 | Honolulu | United States | 0.0262 | Brussels | Belgium | 0.0342 |
| 65 | Shenzhen | China | 0.0258 | Shenzhen | China | 0.0324 |
| 66 | Austin | United States | 0.0248 | Tel-aviv | Israel | 0.0323 |
| 67 | Phnom-penh | Cambodia | 0.0248 | San Diego | United States | 0.0322 |
| 68 | Brussels | Belgium | 0.0239 | Venice | Italy | 0.0314 |
| 69 | Coolangatta | Australia | 0.0237 | Honolulu | United States | 0.0309 |
| 70 | San Diego | United States | 0.0234 | Phnom-penh | Cambodia | 0.0303 |
| 71 | Cairns | Australia | 0.0233 | Norfolk | United States | 0.0299 |
| 72 | Tiruchirappalli | India | 0.0231 | Coolangatta | Australia | 0.0292 |
| 73 | Tel-aviv | Israel | 0.0229 | Osaka | Japan | 0.0291 |
| 74 | Pulau | Malaysia | 0.0222 | Austin | United States | 0.0286 |
| 75 | Norfolk | United States | 0.0220 | Pulau | Malaysia | 0.0285 |
| 76 | Venice | Italy | 0.0217 | Cairns | Australia | 0.0281 |
| 77 | Bandar Seri Begawan | Brunei | 0.0211 | Tiruchirappalli | India | 0.0280 |
| 78 | Tallahassee | United States | 0.0211 | Montreal | Canada | 0.0267 |
| 79 | Macau | Macau | 0.0202 | Luanda | Angola | 0.0264 |
| 80 | Krabi | Thailand | 0.0200 | Macau | Macau | 0.0256 |
| 81 | Osaka | Japan | 0.0197 | Krabi | Thailand | 0.0253 |
| 82 | Fort Myers | United States | 0.0196 | Xiamen | China | 0.0253 |
| 83 | Pensacola | United States | 0.0195 | Bandar Seri Begawan | Brunei | 0.0249 |
| 84 | Xiamen | China | 0.0194 | Duesseldorf | Germany | 0.0248 |

| | | | | | | |
|---|---|---|---|---|---|---|
| 85 | Luanda | Angola | 0.0194 | Tallahassee | United States | 0.0244 |
| 86 | Montreal | Canada | 0.0191 | Raleigh-durham | United States | 0.0234 |
| 87 | Tucson | United States | 0.0188 | Pensacola | United States | 0.0230 |
| 88 | Bermuda | Bermuda | 0.0179 | Fort Myers | United States | 0.0222 |
| 89 | Duesseldorf | Germany | 0.0176 | Beijing | China | 0.0221 |
| 90 | Raleigh-durham | United States | 0.0173 | Bermuda | Bermuda | 0.0209 |
| 91 | Cebu | Philippines | 0.0164 | Zurich | Switzerland | 0.0206 |
| 92 | Gainesville | United States | 0.0163 | Manchester | United Kingdom | 0.0204 |
| 93 | Savannah | United States | 0.0160 | Cebu | Philippines | 0.0203 |
| 94 | Beijing | China | 0.0156 | Tucson | United States | 0.0202 |
| 95 | Sanford | United States | 0.0155 | Berlin | Germany | 0.0187 |
| 96 | Key West | United States | 0.0148 | Gainesville | United States | 0.0182 |
| 97 | Kuching | Malaysia | 0.0146 | Savannah | United States | 0.0181 |
| 98 | Bangalore | India | 0.0146 | San Jose | United States | 0.0181 |
| 99 | Zurich | Switzerland | 0.0144 | Bangalore | India | 0.0176 |
| 100 | Manchester | United Kingdom | 0.0144 | Hamburg | Germany | 0.0176 |
