# Supplementary material for: Vector status of Aedes species determines geographical risk of autochthonous Zika virus establishment
Source: PLoS Negl Trop Dis. 2017 Mar 24;11(3):e0005487. doi: 10.1371/journal.pntd.0005487 (PMC5381944; doi:10.1371/journal.pntd.0005487)
Supplement: S2 Table — (PDF) [file pntd.0005487.s002.pdf]

S2 TABLE: Top 100 Origin Cities Posing Risk under all Scenarios

	Scenario A			Scenario B		
Ranking	City	Country	Relative Risk	City	Country	Relative Risk
1	Singapore	Singapore	0.2635	Singapore	Singapore	0.3159
2	Miami	United States	0.0708	Miami	United States	0.0907
3	San Juan	Puerto Rico	0.0637	San Juan	Puerto Rico	0.0723
4	Cancun	Mexico	0.0482	Cancun	Mexico	0.0580
5	Nadi	Fiji	0.0283	Nadi	Fiji	0.0322
6	Nassau	Bahamas	0.0268	Nassau	Bahamas	0.0291
7	Panama City	Panama	0.0196	Rio De Janeiro	Brazil	0.0223
8	Montego Bay	Jamaica	0.0179	Panama City	Panama	0.0223
9	Rio De Janeiro	Brazil	0.0178	Montego Bay	Jamaica	0.0206
10	Punta Cana	Dominican Republic	0.0153	Buenos Aires	Argentina	0.0204
11	Sao Paulo	Brazil	0.0146	Sao Paulo	Brazil	0.0201
12	Buenos Aires	Argentina	0.0146	Punta Cana	Dominican Republic	0.0185
13	Port Moresby	Papua New Guinea	0.0128	Port Moresby	Papua New Guinea	0.0143
14	St. Thomas	Virgin Islands	0.0114	Santo Domingo	Dominican Republic	0.0140
15	Santo Domingo	Dominican Republic	0.0108	St. Thomas	Virgin Islands	0.0126
16	Port-of-spain	Trinidad and Tobago	0.0095	Port-of-spain	Trinidad and Tobago	0.0109
17	Campinas	Brazil	0.0079	Oranjestad	Aruba	0.0094
18	Oranjestad	Aruba	0.0077	Campinas	Brazil	0.0088
19	Kingston	Jamaica	0.0075	Kingston	Jamaica	0.0087
20	San Jose	Costa Rica	0.0073	San Jose	Costa Rica	0.0087
21	Monterrey	Mexico	0.0062	Monterrey	Mexico	0.0070
22	Noumea	New Caledonia	0.0060	Noumea	New Caledonia	0.0070
23	Port-au-prince	Haiti	0.0059	Port-au-prince	Haiti	0.0068
24	San Jose Del Cabo	Mexico	0.0056	San Jose Del Cabo	Mexico	0.0068
25	San Salvador	El Salvador	0.0053	San Salvador	El Salvador	0.0065

26	Georgetown	Cayman Islands	0.0051	Guadalajara	Mexico	0.0064
27	Aguadilla	Puerto Rico	0.0051	Havana	Cuba	0.0059
28	Guadalajara	Mexico	0.0050	Aguadilla	Puerto Rico	0.0058
29	Freeport	Bahamas	0.0045	Guayaquil	Ecuador	0.0058
30	Guayaquil	Ecuador	0.0044	Georgetown	Cayman Islands	0.0057
31	Philipsburg	Netherlands Antilles	0.0040	Philipsburg	Netherlands Antilles	0.0049
32	San Pedro Sula	Honduras	0.0038	Freeport	Bahamas	0.0049
33	Havana	Cuba	0.0037	San Pedro Sula	Honduras	0.0043
34	Managua	Nicaragua	0.0033	Managua	Nicaragua	0.0038
35	Marsh Harbor	Bahamas	0.0032	Puerto Vallarta	Mexico	0.0037
36	St. Croix Island	Virgin Islands	0.0030	Marsh Harbor	Bahamas	0.0034
37	Ponce	Puerto Rico	0.0029	Salvador	Brazil	0.0033
38	Belize City	Belize	0.0028	Ponce	Puerto Rico	0.0033
39	Puerto Vallarta	Mexico	0.0027	St. Croix Island	Virgin Islands	0.0032
40	Salvador	Brazil	0.0026	Caracas	Venezuela	0.0032
41	Faleolo	Samoa	0.0025	Belize City	Belize	0.0032
42	Caracas	Venezuela	0.0025	Recife	Brazil	0.0031
43	Recife	Brazil	0.0024	Faleolo	Samoa	0.0030
44	Antigua	Antigua and Barbuda	0.0024	Antigua	Antigua and Barbuda	0.0030
45	Brasilia	Brazil	0.0022	Brasilia	Brazil	0.0029
46	Tongatapu	Tonga	0.0020	Willemstad	Curacao	0.0028
47	Liberia	Costa Rica	0.0019	Guatemala City	Guatemala	0.0025
48	Cozumel	Mexico	0.0019	Bridgetown	Barbados	0.0025
49	Guatemala City	Guatemala	0.0019	Bogota	Colombia	0.0025
50	Vera Cruz	Mexico	0.0018	Tongatapu	Tonga	0.0025
51	Bogota	Colombia	0.0018	Liberia	Costa Rica	0.0022
52	Willemstad	Netherlands Antilles	0.0017	Belo Horizonte	Brazil	0.0021
53	Nausori	Fiji	0.0016	Cozumel	Mexico	0.0021
54	Belo Horizonte	Brazil	0.0016	Hewandorra	Saint Lucia	0.0021

55	Hewandorra	Saint Lucia	0.0016	Santiago	Dominican Republic	0.0020
56	Merida	Mexico	0.0015	Vera Cruz	Mexico	0.0019
57	Bridgetown	Barbados	0.0015	Fort-de-france	Martinique	0.0019
58	Villahermosa	Mexico	0.0014	Cartagena	Colombia	0.0018
59	Cartagena	Colombia	0.0014	Nausori	Fiji	0.0018
60	Asuncion	Paraguay	0.0012	Pointe-a-pitre	Guadeloupe	0.0018
61	Ciudad Del Carmen	Mexico	0.0012	Merida	Mexico	0.0017
62	Majuro	Marshall Islands	0.0011	Porto Alegre	Brazil	0.0016
63	Santiago	Dominican Republic	0.0011	Fortaleza	Brazil	0.0016
64	Porto Alegre	Brazil	0.0011	Villahermosa	Mexico	0.0016
65	Providenciales	Turks and Caicos Islands	0.0011	Asuncion	Paraguay	0.0015
66	North Eleuthera	Bahamas	0.0011	Providenciales	Turks and Caicos Islands	0.0014
67	Maceio	Brazil	0.0011	Ciudad Del Carmen	Mexico	0.0013
68	Tampico	Mexico	0.0010	Majuro	Marshall Islands	0.0012
69	Fortaleza	Brazil	0.0010	Natal	Brazil	0.0012
70	Vitoria	Brazil	0.0010	Mexico City	Mexico	0.0012
71	Natal	Brazil	0.0009	Maceio	Brazil	0.0012
72	Fort-de-france	Martinique	0.0009	Vitoria	Brazil	0.0012
73	Pointe-a-pitre	Guadeloupe	0.0009	North Eleuthera	Bahamas	0.0011
74	Tegucigalpa	Honduras	0.0008	Tampico	Mexico	0.0011
75	Alice Town	Bahamas	0.0008	Tegucigalpa	Honduras	0.0010
76	Del Bajio	Mexico	0.0008	Kralendijk	Bonaire	0.0010
77	Georgetown	Guyana	0.0007	Georgetown	Guyana	0.0010
78	Rio Negro	Colombia	0.0007	Del Bajio	Mexico	0.0010
79	Florianopolis	Brazil	0.0007	Rio Negro	Colombia	0.0009
80	Chuuk	Micronesia	0.0007	Curitiba	Brazil	0.0009
81	Mazatlan	Mexico	0.0007	Varadero	Cuba	0.0009

82	Curitiba	Brazil	0.0007	Mazatlan	Mexico	0.0009
83	Yap	Micronesia	0.0007	Florianopolis	Brazil	0.0009
84	Kralendijk	Netherlands Antilles	0.0007	Alice Town	Bahamas	0.0008
85	Basse Terre	Saint Kitts and Nevis	0.0007	Point Salines	Grenada	0.0008
86	Roatan	Honduras	0.0006	Santa Cruz	Bolivia	0.0008
87	Aracaju	Brazil	0.0006	Basse Terre	Saint Kitts and Nevis	0.0008
88	Point Salines	Grenada	0.0006	Chuuk	Micronesia	0.0008
89	Great Exuma	Bahamas	0.0006	Yap	Micronesia	0.0007
90	Santa Cruz	Bolivia	0.0006	Roatan	Honduras	0.0007
91	Manaus	Brazil	0.0005	Aracaju	Brazil	0.0007
92	Culiacan	Mexico	0.0005	Puerto Plata	Dominican Republic	0.0007
93	Puerto Plata	Dominican Republic	0.0005	Great Exuma	Bahamas	0.0007
94	Varadero	Cuba	0.0005	Manaus	Brazil	0.0007
95	Pago Pago	American Samoa	0.0005	Zandery	Suriname	0.0006
96	Mexico City	Mexico	0.0004	Culiacan	Mexico	0.0006
97	Maracaibo	Venezuela	0.0004	Praia, Santiago Island	Cape Verde	0.0005
98	Porto Seguro	Brazil	0.0004	Pago Pago	American Samoa	0.0005
99	Londrina	Brazil	0.0004	Maracaibo	Venezuela	0.0005
100	Goiania	Brazil	0.0003	Goiania	Brazil	0.0005

	Scenario C			Scenario D		
Ranking	City	Country	Relative Risk	City	Country	Relative Risk
1	Singapore	Singapore	0.4034	Singapore	Singapore	0.5727
2	Miami	United States	0.1223	Miami	United States	0.1800
3	San Juan	Puerto Rico	0.0861	San Juan	Puerto Rico	0.1119
4	Cancun	Mexico	0.0734	Cancun	Mexico	0.1007
5	Nadi	Fiji	0.0382	Sao Paulo	Brazil	0.0507
6	Nassau	Bahamas	0.0326	Nadi	Fiji	0.0492
7	Buenos Aires	Argentina	0.0301	Buenos Aires	Argentina	0.0491
8	Sao Paulo	Brazil	0.0300	Rio De Janeiro	Brazil	0.0431
9	Rio De Janeiro	Brazil	0.0296	Nassau	Bahamas	0.0388
10	Panama City	Panama	0.0267	Panama City	Panama	0.0349
11	Montego Bay	Jamaica	0.0250	Punta Cana	Dominican Republic	0.0331
12	Punta Cana	Dominican Republic	0.0238	Montego Bay	Jamaica	0.0331
13	Santo Domingo	Dominican Republic	0.0191	Santo Domingo	Dominican Republic	0.0285
14	Port Moresby	Papua New Guinea	0.0167	Port Moresby	Papua New Guinea	0.0212
15	St. Thomas	Virgin Islands	0.0145	St. Thomas	Virgin Islands	0.0177
16	Port-of-spain	Trinidad and Tobago	0.0132	Port-of-spain	Trinidad and Tobago	0.0173
17	Oranjestad	Aruba	0.0121	Oranjestad	Aruba	0.0172
18	San Jose	Costa Rica	0.0110	Havana	Cuba	0.0157
19	Kingston	Jamaica	0.0107	San Jose	Costa Rica	0.0156
20	Campinas	Brazil	0.0103	Kingston	Jamaica	0.0144
21	Havana	Cuba	0.0093	Guadalajara	Mexico	0.0131
22	Guadalajara	Mexico	0.0087	Campinas	Brazil	0.0130
23	San Jose Del Cabo	Mexico	0.0087	Guayaquil	Ecuador	0.0125
24	San Salvador	El Salvador	0.0085	San Salvador	El Salvador	0.0124
25	Noumea	New Caledonia	0.0085	San Jose Del Cabo	Mexico	0.0120
26	Monterrey	Mexico	0.0083	Noumea	New Caledonia	0.0113

27	Port-au-prince	Haiti	0.0083	Port-au-prince	Haiti	0.0110
28	Guayaquil	Ecuador	0.0080	Monterrey	Mexico	0.0104
29	Aguadilla	Puerto Rico	0.0068	Aguadilla	Puerto Rico	0.0089
30	Georgetown	Cayman Islands	0.0067	Philipsburg	Netherlands Antilles	0.0088
31	Philipsburg	Netherlands Antilles	0.0063	Georgetown	Cayman Islands	0.0085
32	Freeport	Bahamas	0.0055	Puerto Vallarta	Mexico	0.0084
33	Puerto Vallarta	Mexico	0.0053	Willemstad	Netherlands Antilles	0.0077
34	San Pedro Sula	Honduras	0.0052	Bridgetown	Barbados	0.0070
35	Salvador	Brazil	0.0046	Salvador	Brazil	0.0068
36	Managua	Nicaragua	0.0046	Caracas	Venezuela	0.0068
37	Willemstad	Curacao	0.0046	San Pedro Sula	Honduras	0.0068
38	Caracas	Venezuela	0.0044	Freeport	Bahamas	0.0066
39	Recife	Brazil	0.0041	Santiago	Dominican Republic	0.0065
40	Bridgetown	Barbados	0.0041	Bogota	Colombia	0.0064
41	Brasilia	Brazil	0.0040	Brasilia	Brazil	0.0063
42	Antigua	Antigua and Barbuda	0.0039	Fort-de-france	Martinique	0.0062
43	Ponce	Puerto Rico	0.0039	Mexico City	Mexico	0.0061
44	Faleolo	Samoa	0.0038	Recife	Brazil	0.0061
45	Bogota	Colombia	0.0038	Pointe-a-pitre	Guadeloupe	0.0060
46	Belize City	Belize	0.0037	Managua	Nicaragua	0.0060
47	St. Croix Island	Virgin Islands	0.0037	Guatemala City	Guatemala	0.0059
48	Marsh Harbor	Bahamas	0.0037	Antigua	Antigua and Barbuda	0.0055
49	Guatemala City	Guatemala	0.0036	Faleolo	Samoa	0.0053
50	Santiago	Dominican Republic	0.0035	Belo Horizonte	Brazil	0.0051
51	Fort-de-france	Martinique	0.0034	Ponce	Puerto Rico	0.0049
52	Pointe-a-pitre	Guadeloupe	0.0033	Belize City	Belize	0.0047
53	Tongatapu	Tonga	0.0032	Porto Alegre	Brazil	0.0046
54	Belo Horizonte	Brazil	0.0031	Hewandorra	Saint Lucia	0.0045
55	Hewandorra	Saint Lucia	0.0029	Tongatapu	Tonga	0.0045

56	Mexico City	Mexico	0.0027	St. Croix Island	Virgin Islands	0.0044
57	Liberia	Costa Rica	0.0026	Fortaleza	Brazil	0.0043
58	Porto Alegre	Brazil	0.0026	Marsh Harbor	Bahamas	0.0041
59	Fortaleza	Brazil	0.0025	Cartagena	Colombia	0.0038
60	Cartagena	Colombia	0.0025	Liberia	Costa Rica	0.0034
61	Cozumel	Mexico	0.0024	Varadero	Cuba	0.0031
62	Vera Cruz	Mexico	0.0022	Cozumel	Mexico	0.0030
63	Nausori	Fiji	0.0021	Providenciales	Turks and Caicos Islands	0.0028
64	Asuncion	Paraguay	0.0019	Asuncion	Paraguay	0.0028
65	Providenciales	Turks and Caicos Islands	0.0019	Zandery	Suriname	0.0026
66	Merida	Mexico	0.0019	Vera Cruz	Mexico	0.0025
67	Villahermosa	Mexico	0.0017	Natal	Brazil	0.0025
68	Natal	Brazil	0.0017	Nausori	Fiji	0.0025
69	Varadero	Cuba	0.0017	Kralendijk	Netherlands Antilles	0.0024
70	Kralendijk	Bonaire	0.0015	Georgetown	Guyana	0.0023
71	Vitoria	Brazil	0.0015	Curitiba	Brazil	0.0023
72	Georgetown	Guyana	0.0014	Merida	Mexico	0.0022
73	Majuro	Marshall Islands	0.0014	Rio Negro	Colombia	0.0021
74	Ciudad Del Carmen	Mexico	0.0014	Vitoria	Brazil	0.0021
75	Curitiba	Brazil	0.0014	Del Bajio	Mexico	0.0021
76	Maceio	Brazil	0.0014	Villahermosa	Mexico	0.0021
77	Tegucigalpa	Honduras	0.0013	Tegucigalpa	Honduras	0.0019
78	Del Bajio	Mexico	0.0013	Cayenne	French Guiana	0.0019
79	Rio Negro	Colombia	0.0013	Santa Cruz	Bolivia	0.0019
80	Zandery	Suriname	0.0013	Point Salines	Grenada	0.0019
81	Tampico	Mexico	0.0012	Lima	Peru	0.0017
82	North Eleuthera	Bahamas	0.0012	Mazatlan	Mexico	0.0017
83	Point Salines	Grenada	0.0012	Puerto Plata	Dominican Republic	0.0017

84	Santa Cruz	Bolivia	0.0012	Praia, Santiago Island	Cape Verde	0.0017
85	Mazatlan	Mexico	0.0012	Majuro	Marshall Islands	0.0017
86	Florianopolis	Brazil	0.0012	Maceio	Brazil	0.0017
87	Puerto Plata	Dominican Republic	0.0010	Florianopolis	Brazil	0.0017
88	Alice Town	Bahamas	0.0009	Ciudad Del Carmen	Mexico	0.0016
89	Basse Terre	Saint Kitts and Nevis	0.0009	Tampico	Mexico	0.0014
90	Praia, Santiago Island	Cape Verde	0.0009	North Eleuthera	Bahamas	0.0014
91	Cayenne	French Guiana	0.0009	Manaus	Brazil	0.0013
92	Manaus	Brazil	0.0009	Quito	Ecuador	0.0013
93	Chuuk	Micronesia	0.0008	Basse Terre	Saint Kitts and Nevis	0.0012
94	Yap	Micronesia	0.0008	Amilcar Cabral	Cape Verde	0.0012
95	Great Exuma	Bahamas	0.0008	Cali	Colombia	0.0011
96	Roatan	Honduras	0.0008	Goiania	Brazil	0.0011
97	Aracaju	Brazil	0.0008	Alice Town	Bahamas	0.0010
98	Lima	Peru	0.0007	Great Exuma	Bahamas	0.0010
99	Goiania	Brazil	0.0007	Chuuk	Micronesia	0.0010
100	Culiacan	Mexico	0.0007	Roatan	Honduras	0.0010

	Scenario E			Scenario F		
Ranking	City	Country	Relative Risk	City	Country	Relative Risk
1	Singapore	Singapore	0.7716	Singapore	Singapore	1.0000
2	Miami	United States	0.2440	Miami	United States	0.3143
3	San Juan	Puerto Rico	0.1411	San Juan	Puerto Rico	0.1736
4	Cancun	Mexico	0.1302	Cancun	Mexico	0.1618
5	Sao Paulo	Brazil	0.0768	Sao Paulo	Brazil	0.1082
6	Buenos Aires	Argentina	0.0715	Buenos Aires	Argentina	0.0974
7	Nadi	Fiji	0.0612	Rio De Janeiro	Brazil	0.0749
8	Rio De Janeiro	Brazil	0.0582	Nadi	Fiji	0.0742
9	Nassau	Bahamas	0.0453	Punta Cana	Dominican Republic	0.0545
10	Panama City	Panama	0.0440	Panama City	Panama	0.0542
11	Punta Cana	Dominican Republic	0.0434	Nassau	Bahamas	0.0522
12	Montego Bay	Jamaica	0.0420	Montego Bay	Jamaica	0.0517
13	Santo Domingo	Dominican Republic	0.0391	Santo Domingo	Dominican Republic	0.0509
14	Port Moresby	Papua New Guinea	0.0261	Port Moresby	Papua New Guinea	0.0316
15	Oranjestad	Aruba	0.0230	Havana	Cuba	0.0306
16	Havana	Cuba	0.0227	Oranjestad	Aruba	0.0294
17	Port-of-spain	Trinidad and Tobago	0.0218	San Jose	Costa Rica	0.0271
18	St. Thomas	Virgin Islands	0.0212	Port-of-spain	Trinidad and Tobago	0.0268
19	San Jose	Costa Rica	0.0209	St. Thomas	Virgin Islands	0.0249
20	Kingston	Jamaica	0.0185	Guayaquil	Ecuador	0.0242
21	Guadalajara	Mexico	0.0181	Guadalajara	Mexico	0.0237
22	Guayaquil	Ecuador	0.0179	Kingston	Jamaica	0.0232
23	San Salvador	El Salvador	0.0168	San Salvador	El Salvador	0.0218
24	Campinas	Brazil	0.0159	San Jose Del Cabo	Mexico	0.0194
25	San Jose Del Cabo	Mexico	0.0156	Campinas	Brazil	0.0192
26	Noumea	New Caledonia	0.0144	Noumea	New Caledonia	0.0178

27	Port-au-prince	Haiti	0.0142	Port-au-prince	Haiti	0.0178
28	Monterrey	Mexico	0.0128	Mexico City	Mexico	0.0163
29	Puerto Vallarta	Mexico	0.0119	Puerto Vallarta	Mexico	0.0160
30	Philipsburg	Netherlands Antilles	0.0115	Monterrey	Mexico	0.0153
31	Willemstad	Netherlands Antilles	0.0112	Willemstad	Netherlands Antilles	0.0149
32	Aguadilla	Puerto Rico	0.0111	Philipsburg	Netherlands Antilles	0.0144
33	Mexico City	Mexico	0.0107	Santiago	Dominican Republic	0.0139
34	Georgetown	Cayman Islands	0.0104	Bridgetown	Barbados	0.0139
35	Bridgetown	Barbados	0.0103	Aguadilla	Puerto Rico	0.0137
36	Santiago	Dominican Republic	0.0099	Bogota	Colombia	0.0135
37	Bogota	Colombia	0.0096	Caracas	Venezuela	0.0126
38	Caracas	Venezuela	0.0095	Pointe-a-pitre	Guadeloupe	0.0126
39	Salvador	Brazil	0.0093	Georgetown	Cayman Islands	0.0125
40	Fort-de-france	Martinique	0.0092	Fort-de-france	Martinique	0.0124
41	Pointe-a-pitre	Guadeloupe	0.0091	Brasilia	Brazil	0.0123
42	Brasilia	Brazil	0.0091	Guatemala City	Guatemala	0.0122
43	Guatemala City	Guatemala	0.0088	Salvador	Brazil	0.0121
44	San Pedro Sula	Honduras	0.0085	San Pedro Sula	Honduras	0.0105
45	Recife	Brazil	0.0082	Belo Horizonte	Brazil	0.0105
46	Freeport	Bahamas	0.0078	Recife	Brazil	0.0105
47	Managua	Nicaragua	0.0076	Porto Alegre	Brazil	0.0104
48	Belo Horizonte	Brazil	0.0076	Managua	Nicaragua	0.0094
49	Porto Alegre	Brazil	0.0072	Freeport	Bahamas	0.0091
50	Antigua	Antigua and Barbuda	0.0071	Antigua	Antigua and Barbuda	0.0089
51	Faleolo	Samoa	0.0070	Faleolo	Samoa	0.0089
52	Hewandorra	Saint Lucia	0.0063	Fortaleza	Brazil	0.0085
53	Fortaleza	Brazil	0.0063	Hewandorra	Saint Lucia	0.0084
54	Ponce	Puerto Rico	0.0061	Tongatapu	Tonga	0.0077
55	Tongatapu	Tonga	0.0060	Ponce	Puerto Rico	0.0074

56	Belize City	Belize	0.0059	Cartagena	Colombia	0.0073
57	Cartagena	Colombia	0.0054	Belize City	Belize	0.0071
58	St. Croix Island	Virgin Islands	0.0052	Varadero	Cuba	0.0063
59	Varadero	Cuba	0.0046	Zandery	Suriname	0.0061
60	Marsh Harbor	Bahamas	0.0046	St. Croix Island	Virgin Islands	0.0060
61	Liberia	Costa Rica	0.0043	Liberia	Costa Rica	0.0052
62	Zandery	Suriname	0.0042	Lima	Peru	0.0051
63	Providenciales	Turks and Caicos Islands	0.0038	Marsh Harbor	Bahamas	0.0051
64	Asuncion	Paraguay	0.0038	Providenciales	Turks and Caicos Islands	0.0050
65	Cozumel	Mexico	0.0036	Asuncion	Paraguay	0.0048
66	Natal	Brazil	0.0034	Curitiba	Brazil	0.0048
67	Curitiba	Brazil	0.0034	Cayenne	French Guiana	0.0048
68	Kralendijk	Netherlands Antilles	0.0034	Georgetown	Guyana	0.0045
69	Georgetown	Guyana	0.0033	Natal	Brazil	0.0044
70	Lima	Peru	0.0032	Kralendijk	Netherlands Antilles	0.0044
71	Cayenne	French Guiana	0.0032	Cozumel	Mexico	0.0043
72	Rio Negro	Colombia	0.0031	Rio Negro	Colombia	0.0043
73	Nausori	Fiji	0.0030	Praia, Santiago Island	Cape Verde	0.0041
74	Vera Cruz	Mexico	0.0030	Del Bajio	Mexico	0.0040
75	Del Bajio	Mexico	0.0029	Amilcar Cabral	Cape Verde	0.0039
76	Vitoria	Brazil	0.0028	Santa Cruz	Bolivia	0.0036
77	Praia, Santiago Island	Cape Verde	0.0028	Vitoria	Brazil	0.0036
78	Santa Cruz	Bolivia	0.0027	Nausori	Fiji	0.0036
79	Tegucigalpa	Honduras	0.0027	Tegucigalpa	Honduras	0.0035
80	Merida	Mexico	0.0026	Puerto Plata	Dominican Republic	0.0034
81	Point Salines	Grenada	0.0026	Point Salines	Grenada	0.0034
82	Puerto Plata	Dominican Republic	0.0025	Quito	Ecuador	0.0034
83	Villahermosa	Mexico	0.0024	Vera Cruz	Mexico	0.0034
84	Amilcar Cabral	Cape Verde	0.0023	Merida	Mexico	0.0030

85	Mazatlan	Mexico	0.0023	Florianopolis	Brazil	0.0030
86	Florianopolis	Brazil	0.0023	Mazatlan	Mexico	0.0029
87	Quito	Ecuador	0.0022	Villahermosa	Mexico	0.0028
88	Maceio	Brazil	0.0020	Cali	Colombia	0.0027
89	Majuro	Marshall Islands	0.0020	Maceio	Brazil	0.0024
90	Ciudad Del Carmen	Mexico	0.0019	Majuro	Marshall Islands	0.0024
91	Cali	Colombia	0.0018	Manaus	Brazil	0.0023
92	Manaus	Brazil	0.0018	Goiania	Brazil	0.0023
93	Tampico	Mexico	0.0017	Ciudad Del Carmen	Mexico	0.0021
94	Goiania	Brazil	0.0016	Holguin	Cuba	0.0019
95	North Eleuthera	Bahamas	0.0015	Tampico	Mexico	0.0019
96	Basse Terre	Saint Kitts and Nevis	0.0015	Cordoba	Argentina	0.0018
97	Holguin	Cuba	0.0014	Aguascalientes	Mexico	0.0018
98	Aguascalientes	Mexico	0.0014	Basse Terre	Saint Kitts and Nevis	0.0018
99	Cordoba	Argentina	0.0012	North Eleuthera	Bahamas	0.0016
100	Great Exuma	Bahamas	0.0012	Belem	Brazil	0.0015
