# Supplementary material for: Vector status of Aedes species determines geographical risk of autochthonous Zika virus establishment
Source: PLoS Negl Trop Dis. 2017 Mar 24;11(3):e0005487. doi: 10.1371/journal.pntd.0005487 (PMC5381944; doi:10.1371/journal.pntd.0005487)
Supplement: S3 Table — (PDF) [file pntd.0005487.s003.pdf]

S3 TABLE: Top 100 High Risk Travel Routes under Scenario A

Ranking	Origin City	Origin Country	Destination City	Destination Country	Relative Risk
1	Singapore	Singapore	Bangkok	Thailand	0.574
2	Singapore	Singapore	Hong Kong	Hong Kong	0.436
3	San Juan	Puerto Rico	Orlando	United States	0.389
4	Singapore	Singapore	Manila	Philippines	0.255
5	San Juan	Puerto Rico	Fort Lauderdale	United States	0.233
6	Nassau	Bahamas	Fort Lauderdale	United States	0.224
7	Singapore	Singapore	Ho Chi Minh City	Vietnam	0.22
8	Singapore	Singapore	Jakarta	Indonesia	0.189
9	Singapore	Singapore	Denpasar	Indonesia	0.176
10	Miami	United States	Houston	United States	0.174
11	Nadi	Fiji	Sydney	Australia	0.145
12	Panama City	Panama	Orlando	United States	0.139
13	Cancun	Mexico	Houston	United States	0.121
14	San Juan	Puerto Rico	Tampa	United States	0.118
15	Cancun	Mexico	Fort Lauderdale	United States	0.112
16	Singapore	Singapore	Phuket	Thailand	0.107
17	Cancun	Mexico	Orlando	United States	0.102
18	Singapore	Singapore	Sydney	Australia	0.096
19	Montego Bay	Jamaica	Fort Lauderdale	United States	0.096
20	Miami	United States	Orlando	United States	0.094
21	Nassau	Bahamas	Orlando	United States	0.094
22	Sao Paulo	Brazil	Orlando	United States	0.092
23	Port Moresby	Papua New Guinea	Brisbane	Australia	0.091
24	Miami	United States	Tampa	United States	0.09
25	Port-of-spain	Trinidad and Tobago	Fort Lauderdale	United States	0.088
26	Nadi	Fiji	Brisbane	Australia	0.086
27	Singapore	Singapore	Madras	India	0.085
28	Singapore	Singapore	Hanoi	Vietnam	0.083
29	Rio De Janeiro	Brazil	Orlando	United States	0.083
30	Singapore	Singapore	Kuala Lumpur	Malaysia	0.08
31	Montego Bay	Jamaica	Orlando	United States	0.075
32	Kingston	Jamaica	Fort Lauderdale	United States	0.075
33	Singapore	Singapore	Guangzhou	China	0.073
34	Singapore	Singapore	Taipei	Taiwan	0.073
35	Punta Cana	Dominican Republic	Orlando	United States	0.071
36	Port-au-prince	Haiti	Fort Lauderdale	United States	0.07
37	Santo Domingo	Dominican Republic	Fort Lauderdale	United States	0.069
38	Singapore	Singapore	Brisbane	Australia	0.068
39	Singapore	Singapore	Surabaya	Indonesia	0.068
40	Buenos Aires	Argentina	Montevideo	Uruguay	0.065
41	Singapore	Singapore	Dhaka	Bangladesh	0.063
42	Campinas	Brazil	Orlando	United States	0.061

**43**	Singapore	Singapore	Yangon	Burma	0.059
**44**	Freeport	Bahamas	Fort Lauderdale	United States	0.059
**45**	St. Thomas	Virgin Islands	Fort Lauderdale	United States	0.058
**46**	Miami	United States	Montevideo	Uruguay	0.055
**47**	Miami	United States	New Orleans	United States	0.054
**48**	Oranjestad	Aruba	Orlando	United States	0.052
**49**	Singapore	Singapore	Mumbai	India	0.052
**50**	Singapore	Singapore	Delhi	India	0.049
**51**	Campinas	Brazil	Fort Lauderdale	United States	0.049
**52**	Aguadilla	Puerto Rico	Orlando	United States	0.049
**53**	Punta Cana	Dominican Republic	Fort Lauderdale	United States	0.048
**54**	Singapore	Singapore	Colombo	Sri Lanka	0.047
**55**	Rio De Janeiro	Brazil	Montevideo	Uruguay	0.047
**56**	Monterrey	Mexico	Houston	United States	0.047
**57**	San Jose	Costa Rica	Fort Lauderdale	United States	0.042
**58**	Ponce	Puerto Rico	Orlando	United States	0.042
**59**	San Juan	Puerto Rico	Houston	United States	0.039
**60**	Singapore	Singapore	Bandar Seri Begawan	Brunei	0.039
**61**	Singapore	Singapore	Phnom-penh	Cambodia	0.039
**62**	Singapore	Singapore	Tiruchirappalli	India	0.038
**63**	Cancun	Mexico	Sanford	United States	0.037
**64**	Nadi	Fiji	Auckland	New Zealand	0.036
**65**	St. Thomas	Virgin Islands	Orlando	United States	0.035
**66**	Singapore	Singapore	Shenzhen	China	0.035
**67**	Georgetown	Cayman Islands	Tampa	United States	0.035
**68**	Port Moresby	Papua New Guinea	Cairns	Australia	0.033
**69**	Singapore	Singapore	Penang	Malaysia	0.033
**70**	San Salvador	El Salvador	Houston	United States	0.032
**71**	Miami	United States	Jacksonville	United States	0.031
**72**	Santo Domingo	Dominican Republic	Orlando	United States	0.031
**73**	San Jose Del Cabo	Mexico	Houston	United States	0.028
**74**	Singapore	Singapore	Pulau	Malaysia	0.027
**75**	Singapore	Singapore	Krabi	Thailand	0.027
**76**	Singapore	Singapore	Macau	Macau	0.027
**77**	Singapore	Singapore	Kuching	Malaysia	0.027
**78**	Guayaquil	Ecuador	Fort Lauderdale	United States	0.027
**79**	San Jose	Costa Rica	Orlando	United States	0.026
**80**	Panama City	Panama	Houston	United States	0.026
**81**	Panama City	Panama	Fort Lauderdale	United States	0.026
**82**	Cancun	Mexico	New Orleans	United States	0.024
**83**	Brasilia	Brazil	Orlando	United States	0.024
**84**	Sao Paulo	Brazil	Montevideo	Uruguay	0.024
**85**	Singapore	Singapore	Cebu	Philippines	0.024
**86**	Miami	United States	Bermuda	Bermuda	0.023
**87**	Miami	United States	Tallahassee	United States	0.022

**88**	Nadi	Fiji	Hong Kong	Hong Kong	0.022
**89**	Buenos Aires	Argentina	Orlando	United States	0.022
**90**	Miami	United States	Charleston	United States	0.022
**91**	Marsh Harbor	Bahamas	Fort Lauderdale	United States	0.022
**92**	Singapore	Singapore	Angeles City	Philippines	0.021
**93**	Marsh Harbor	Bahamas	West Palm Beach	United States	0.021
**94**	Noumea	New Caledonia	Sydney	Australia	0.021
**95**	Singapore	Singapore	Xiamen	China	0.021
**96**	Singapore	Singapore	Kolkata	India	0.021
**97**	Singapore	Singapore	Kaohsiung	Taiwan	0.021
**98**	Cancun	Mexico	Fort Myers	United States	0.021
**99**	Aguadilla	Puerto Rico	Fort Lauderdale	United States	0.02
**100**	St. Thomas	Virgin Islands	Tampa	United States	0.02
