# Supplementary material for: Vector status of Aedes species determines geographical risk of autochthonous Zika virus establishment
Source: PLoS Negl Trop Dis. 2017 Mar 24;11(3):e0005487. doi: 10.1371/journal.pntd.0005487 (PMC5381944; doi:10.1371/journal.pntd.0005487)
Supplement: S4 Table — (PDF) [file pntd.0005487.s004.pdf]

S4 TABLE: Top 100 High Risk Travel Routes under Scenario D

Ranking	Origin City	Origin Country	Destination City	Destination Country	Relative Risk
1	Singapore	Singapore	Bangkok	Thailand	1
2	Singapore	Singapore	Hong Kong	Hong Kong	0.92
3	Singapore	Singapore	Jakarta	Indonesia	0.681
4	San Juan	Puerto Rico	Orlando	United States	0.51
5	Singapore	Singapore	Manila	Philippines	0.478
6	Singapore	Singapore	Kuala Lumpur	Malaysia	0.449
7	Singapore	Singapore	Ho Chi Minh City	Vietnam	0.377
8	Singapore	Singapore	Denpasar	Indonesia	0.364
9	Miami	United States	New York	United States	0.351
10	San Juan	Puerto Rico	Fort Lauderdale	United States	0.318
11	Singapore	Singapore	Tokyo	Japan	0.302
12	Singapore	Singapore	Shanghai	China	0.232
13	Nassau	Bahamas	Fort Lauderdale	United States	0.217
14	Singapore	Singapore	Taipei	Taiwan	0.21
15	Singapore	Singapore	Phuket	Thailand	0.209
16	Nadi	Fiji	Sydney	Australia	0.204
17	Singapore	Singapore	Sydney	Australia	0.199
18	Singapore	Singapore	Guangzhou	China	0.18
19	Sao Paulo	Brazil	Orlando	United States	0.179
20	Miami	United States	Houston	United States	0.171
21	Singapore	Singapore	Seoul	South Korea	0.161
22	San Juan	Puerto Rico	Tampa	United States	0.161
23	Panama City	Panama	Orlando	United States	0.16
24	Singapore	Singapore	Penang	Malaysia	0.152
25	San Juan	Puerto Rico	New York	United States	0.142
26	Singapore	Singapore	Surabaya	Indonesia	0.141
27	Singapore	Singapore	Hanoi	Vietnam	0.138
28	Port Moresby	Papua New Guinea	Brisbane	Australia	0.13
29	Singapore	Singapore	Perth	Australia	0.128
30	Singapore	Singapore	Madras	India	0.126
31	Buenos Aires	Argentina	Montevideo	Uruguay	0.126
32	Singapore	Singapore	Mumbai	India	0.126
33	Singapore	Singapore	Yangon	Burma	0.123
34	Singapore	Singapore	Brisbane	Australia	0.12
35	Nadi	Fiji	Auckland	New Zealand	0.115
36	Cancun	Mexico	Fort Lauderdale	United States	0.112
37	Singapore	Singapore	Dhaka	Bangladesh	0.111
38	Santo Domingo	Dominican Republic	New York	United States	0.111
39	Cancun	Mexico	Houston	United States	0.11
40	Miami	United States	Washington	United States	0.11
41	Cancun	Mexico	New York	United States	0.107
42	Montego Bay	Jamaica	Fort Lauderdale	United States	0.106

**43**	Buenos Aires	Argentina	Santiago	Chile	0.104
**44**	Miami	United States	Newark	United States	0.103
**45**	Nadi	Fiji	Brisbane	Australia	0.102
**46**	Port-au-prince	Haiti	Fort Lauderdale	United States	0.1
**47**	Cancun	Mexico	Orlando	United States	0.098
**48**	Miami	United States	Orlando	United States	0.098
**49**	Port-of-spain	Trinidad and Tobago	Fort Lauderdale	United States	0.097
**50**	Miami	United States	Tampa	United States	0.096
**51**	Miami	United States	Atlanta	United States	0.093
**52**	Miami	United States	Los Angeles	United States	0.093
**53**	Kingston	Jamaica	Fort Lauderdale	United States	0.092
**54**	Singapore	Singapore	Colombo	Sri Lanka	0.09
**55**	Rio De Janeiro	Brazil	Orlando	United States	0.089
**56**	Nassau	Bahamas	Orlando	United States	0.087
**57**	Willemstad	Netherlands Antilles	Amsterdam	Netherlands	0.087
**58**	Miami	United States	Montevideo	Uruguay	0.086
**59**	Campinas	Brazil	Orlando	United States	0.085
**60**	Miami	United States	Philadelphia	United States	0.081
**61**	Miami	United States	San Francisco	United States	0.081
**62**	Montego Bay	Jamaica	Orlando	United States	0.079
**63**	Miami	United States	New Orleans	United States	0.079
**64**	Miami	United States	London	United Kingdom	0.079
**65**	Santo Domingo	Dominican Republic	Fort Lauderdale	United States	0.078
**66**	Buenos Aires	Argentina	New York	United States	0.077
**67**	Rio De Janeiro	Brazil	Montevideo	Uruguay	0.076
**68**	Santiago	Dominican Republic	New York	United States	0.074
**69**	Singapore	Singapore	Shenzhen	China	0.072
**70**	Fort-de-france	Martinique	Paris	France	0.072
**71**	Cancun	Mexico	Los Angeles	United States	0.072
**72**	Punta Cana	Dominican Republic	Orlando	United States	0.071
**73**	Campinas	Brazil	Fort Lauderdale	United States	0.071
**74**	Sao Paulo	Brazil	New York	United States	0.071
**75**	Sao Paulo	Brazil	Montevideo	Uruguay	0.07
**76**	Buenos Aires	Argentina	Rome	Italy	0.07
**77**	Pointe-a-pitre	Guadeloupe	Paris	France	0.069
**78**	Singapore	Singapore	Phnom-penh	Cambodia	0.068
**79**	Aguadilla	Puerto Rico	Orlando	United States	0.065
**80**	San Jose	Costa Rica	Fort Lauderdale	United States	0.065
**81**	Singapore	Singapore	Pulau	Malaysia	0.064
**82**	Cancun	Mexico	London	United Kingdom	0.063
**83**	Singapore	Singapore	Tiruchirappalli	India	0.063
**84**	Singapore	Singapore	Delhi	India	0.063
**85**	Bridgetown	Barbados	London	United Kingdom	0.062
**86**	Freeport	Bahamas	Fort Lauderdale	United States	0.061
**87**	Singapore	Singapore	London	United Kingdom	0.06

**88**	Oranjestad	Aruba	Orlando	United States	0.058
**89**	St. Thomas	Virgin Islands	Fort Lauderdale	United States	0.058
**90**	Singapore	Singapore	Macau	Macau	0.058
**91**	Singapore	Singapore	Krabi	Thailand	0.057
**92**	Guayaquil	Ecuador	New York	United States	0.057
**93**	Guadalajara	Mexico	Los Angeles	United States	0.057
**94**	Singapore	Singapore	Melbourne	Australia	0.057
**95**	Sao Paulo	Brazil	Lisbon	Portugal	0.056
**96**	Singapore	Singapore	Bandar Seri Begawan	Brunei	0.056
**97**	Ponce	Puerto Rico	Orlando	United States	0.056
**98**	Buenos Aires	Argentina	Barcelona	Spain	0.055
**99**	Port-of-spain	Trinidad and Tobago	New York	United States	0.054
**100**	Rio De Janeiro	Brazil	Lisbon	Portugal	0.054
