# Supplementary material for: Vector status of Aedes species determines geographical risk of autochthonous Zika virus establishment
Source: PLoS Negl Trop Dis. 2017 Mar 24;11(3):e0005487. doi: 10.1371/journal.pntd.0005487 (PMC5381944; doi:10.1371/journal.pntd.0005487)
Supplement: S5 Table — (PDF) [file pntd.0005487.s005.pdf]

S5 TABLE: Set of Countries with local Zika transmission confirmed between February 15 and October 5 and relative ranking by Scenario.

	Country	Rank in Scenario A	Rank in Scenario B	Rank in Scenario C	Rank in Scenario D	Rank in Scenario E	Rank in Scenario F
1	United States	**1**	**1**	**1**	**1**	**1**	**1**
2	Argentina	**2**	**2**	**2**	**2**	**2**	**2**
3	Cuba	**3**	**3**	**3**	**3**	**3**	**3**
4	Aruba	**4**	**4**	**4**	**4**	**4**	**4**
5	Bahamas	**5**	**5**	**5**	**5**	**5**	**8**
6	Curacao	**6**	**6**	**6**	**6**	**6**	**7**
7	Trinidad and Tobago	**7**	**7**	**7**	**7**	**7**	**11**
8	Cayman Islands	**9**	**9**	**9**	**14**	**14**	**16**
9	Saint Martin	**10**	**10**	**10**	**15**	**16**	**19**
10	Saint Barts	**11**	**12**	**16**	**18**	**22**	**22**
11	British Virgin Islands	**12**	**11**	**15**	**16**	**20**	**21**
12	Antigua and Barbuda	**13**	**13**	**19**	**23**	**23**	**25**
13	Saint Kitts and Nevis	**14**	**14**	**20**	**24**	**24**	**28**
14	Belize	**16**	**17**	**21**	**25**	**25**	**26**
15	Turks and Caicos Islands	**17**	**18**	**22**	**26**	**26**	30
16	Saint Lucia	**18**	**20**	**23**	**27**	**28**	31
17	Saba	**22**	**26**	33	36	41	43
18	Grenada	**25**	**29**	34	35	38	40
19	Bonaire	**27**	31	35	38	43	46
20	Anguilla	**28**	33	38	43	47	48
21	Dominica	**29**	35	39	41	44	44
22	Fiji	31	38	40	44	45	45
23	Saint Vincent and the Grenadines	37	42	45	50	52	54
24	Singapore	52	57	60	61	60	60
25	Peru	53	45	41	33	31	**29**
26	New Caledonia	97	108	111	117	118	119
27	Papua New Guinea	106	115	118	122	125	128
28	Federated States of Micronesia	#N/A	#N/A	#N/A	#N/A	#N/A	#N/A
29	Marshall Islands	#N/A	#N/A	#N/A	#N/A	#N/A	#N/A
