# Supplementary material for: Vector status of Aedes species determines geographical risk of autochthonous Zika virus establishment
Source: PLoS Negl Trop Dis. 2017 Mar 24;11(3):e0005487. doi: 10.1371/journal.pntd.0005487 (PMC5381944; doi:10.1371/journal.pntd.0005487)
Supplement: S6 Table — (PDF) [file pntd.0005487.s006.pdf]

S6 TABLE: Top 29 Countries identified at greatest risk by each scenario based on outbreak status as of February 15, 2016

Rank	Scenario A	Scenario B	Scenario C	Scenario D	Scenario E	Scenario F
1	United States	United States	United States	United States	United States	United States
2	Argentina	Argentina	Argentina	Argentina	Argentina	Argentina
3	Cuba	Cuba	Cuba	Cuba	Cuba	Cuba
4	Aruba	Aruba	Aruba	Aruba	Aruba	Aruba
5	Bahamas	Bahamas	Bahamas	Bahamas	Bahamas	Italy
6	Curacao	Curacao	Curacao	Curacao	Curacao	France
7	Trinidad and Tobago	Trinidad and Tobago	Trinidad and Tobago	Trinidad and Tobago	Trinidad and Tobago	Curacao
8	Uruguay	Uruguay	Uruguay	Uruguay	Uruguay	Bahamas
9	Cayman Islands	Cayman Islands	Cayman Islands	Italy	Italy	Portugal
10	Saint Martin	Saint Martin	Saint Martin	France	France	Uruguay
11	Saint Barts	British Virgin Islands	Italy	Portugal	Portugal	Trinidad and Tobago
12	British Virgin Islands	Saint Barts	France	Spain	United Kingdom	United Kingdom
13	Antigua and Barbuda	Antigua and Barbuda	Spain	United Kingdom	Spain	Spain
14	Saint Kitts and Nevis	Saint Kitts and Nevis	Portugal	Cayman Islands	Cayman Islands	Chile
15	Australia	Australia	British Virgin Islands	Saint Martin	Chile	Germany
16	Belize	Italy	Saint Barts	British Virgin Islands	Saint Martin	Cayman Islands
17	Turks and Caicos Islands	Belize	United Kingdom	Chile	Germany	Canada
18	Saint Lucia	Turks and Caicos Islands	Australia	Saint Barts	Canada	Netherlands
19	Spain	Spain	Antigua and Barbuda	Australia	Netherlands	Saint Martin
20	India	Saint Lucia	Saint Kitts and Nevis	Germany	British Virgin Islands	Australia
21	Italy	Portugal	Belize	Canada	Australia	British Virgin Islands
22	Saba	France	Turks and Caicos Islands	Netherlands	Saint Barts	Saint Barts
23	Portugal	United Kingdom	Saint Lucia	Antigua and Barbuda	Antigua and Barbuda	Japan
24	Hong Kong	India	Chile	Saint Kitts and Nevis	Saint Kitts and Nevis	China
25	Grenada	Hong Kong	Canada	Belize	Belize	Antigua and Barbuda
26	Philippines	Saba	Germany	Turks and Caicos Islands	Turks and Caicos Islands	Belize
27	Bonaire	Chile	Netherlands	Saint Lucia	Japan	New Zealand
28	Anguilla	Philippines	India	New Zealand	Saint Lucia	Saint Kitts and Nevis
29	Dominica	Grenada	Hong Kong	China	China	Peru
